# Disruption of Bile Acid Metabolism in the Gut–Liver Axis Predisposes Mice to Inflammatory Bowel Disease

**DOI:** 10.1002/mco2.70429

**Published:** 2025-10-09

**Authors:** Hui Chang, Yang Jiang, Qiong Zhao, Zhen Su, Mingyang Chen, Qiufen He, Jingbo Lai, Yingru Jiang, Jing Zheng, Ruolang Pan, Jianzhong Shao, Robert Chunhua Zhao, Ye Chen

**Affiliations:** ^1^ College of Life Sciences and Department of Genetic and Metabolic Disease The Children's Hospital Zhejiang University School of Medicine National Clinical Research Center for Child Health Zhejiang University Hangzhou China; ^2^ College of Pharmaceutical Sciences Zhejiang University Hangzhou China; ^3^ Reproductive Medical Center The First Affiliated Hospital Zhejiang University School of Medicine Hangzhou China; ^4^ Institute For Cell‐Based Drug Development of Zhejiang Province Hangzhou China; ^5^ Institute of Basic Medical Sciences Chinese Academy of Medical Sciences School of Basic Medicine Peking Union Medical College Beijing China

**Keywords:** primary sclerosing cholangitis, bile acid, gut–liver axis, immune cell homeostasis, susceptibility to IBD

## Abstract

Primary sclerosing cholangitis (PSC) is a chronic cholestatic liver disease that is frequently associated with inflammatory bowel disease (IBD). However, the precise mechanisms linking these conditions remain unclear. In this study, we established a murine model of experimental sclerosing cholangitis (eSC) using a DDC (3,5‐diethoxycarbonyl 1,4‐dihydrocollidine) diet. We then demonstrated that eSC mice exhibited increased susceptibility to DSS‐induced colitis, accompanied by severe intestinal pathology. Further integrated analyses revealed that eSC disrupted bile acid metabolism and gut microbiota composition, notably increasing Th17‐inducing bacteria and altering bile acid profiles. Single‐cell and bulk RNA‐seq analyses identified a marked expansion of colonic Th17 cells and a loss of immune homeostasis in eSC mice. Therapeutically, rectal administration of lithocholic acid (LCA) and its derivative, 3‐Oxo‐5β‐cholanoic acid (3‐O‐LCA), was found to restore farnesoid X receptor (FXR) signaling, reduce Th17 cell proportions, and alleviate liver and intestinal injury. Mechanistic studies show that LCA and 3‐O‐LCA modulate macrophage polarization and Th17 differentiation via FXR. These findings highlight the central role of the gut–liver axis, bile acid signaling, and Th17 responses in PSC–IBD pathogenesis, and suggest that targeting bile acid metabolism offers a promising therapeutic strategy. This work advances our understanding of PSC–IBD and provides a foundation for novel interventions in high‐risk patients.

## Graphical abstract

An imbalance among bile acids, immune hemostasis, and microbes potentially contributes to increased susceptibility to IBD in a mouse model of PSC. This disruption can be mitigated by the administration of LCA and 3‐O‐LCA. Mechanistically, LCA and 3‐O‐LCA can antagonize T‐β‐MCA‐induced macrophage FXR inhibition and the resulting enhancement of Th17 differentiation.

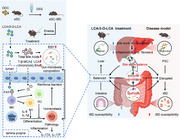

## 1 Introduction

Primary sclerosing cholangitis (PSC) is a chronic liver disease characterized by inflammation and scarring of the bile ducts, ultimately leading to biliary cirrhosis and liver failure [1, 2]. The etiology of PSC remains incompletely elucidated, although multiple factors, such as genetic predisposition, immune dysregulation, and gut microbiome alterations, are known to contribute to its pathogenesis [3, 4]. Notably, PSC is strongly associated with inflammatory bowel disease (IBD), with approximately 50%–80% of PSC patients also diagnosed with IBD, predominantly ulcerative colitis [5]. Moreover, PSC–IBD patients exhibit distinct clinical features and are at increased risk for colorectal cancer and cholangiocarcinoma, underscoring the urgency of elucidating the mechanisms linking these two diseases [6, 7].

The “gut–liver axis” concept was first introduced in 1998 by Marshall et al. [8], highlighting the bidirectional communication between the gut and liver through the portal vein, which supplies approximately 70% of the blood to the liver. Intestinal epithelial cells and an accompanying mucous layer form a barrier that prevents harmful substances from entering the bloodstream. Disruption of this barrier during intestinal inflammation permits the translocation of bacterial products and microbial metabolites into the portal vein, resulting in hepatic immune activation [9, 10]. Moreover, gut‐primed lymphocytes expressing specific homing receptors can migrate to the liver, perpetuating inflammation even in the absence of active gut disease [11]. IBD arises from a complex interplay of genetic susceptibility, dysregulated immune responses, environmental triggers, and gut microbiota alterations [12, 13, 14, 15].

Overall, intestinal barrier dysfunction and microbial dysbiosis are known to increase exposure of the immune system to luminal antigens, thereby promoting chronic inflammation. Interestingly, the gut microbiota of patients with PSC–IBD is distinct from the gut microbiota in patients with IBD alone; this difference is characterized by reduced diversity and enrichment of proinflammatory taxa [16], and may intensify both intestinal and hepatic function. The “gut–liver axis” provides insights into liver disease inflammation. However, the disruption of gut homeostasis may be a critical factor linking PSC and IBD. These mechanisms provide a plausible explanation for the close association of PSC and IBD, as well as the persistence of hepatic inflammation regardless of intestinal activity.

Bile acids, synthesized from cholesterol in the liver and conjugated with glycine or taurine, are stored in the gallbladder and released into the duodenum during digestion [17]. Gut bacteria convert these bile acids into secondary derivatives like deoxycholic acid (DCA) and lithocholic acid (LCA) [18]. Approximately 95% of bile acids are reabsorbed in the ileum and transported back to the liver via enterohepatic circulation [19, 20]. Beyond their classical role in lipid digestion, bile acids act as signaling molecules capable of regulating metabolic and immune pathways via receptors such as farnesoid X receptor (FXR) and G protein‐coupled bile acid receptor 1 (TGR5). Dysregulation of bile acid metabolism has been implicated in numerous conditions, including alcoholic liver disease [21], nonalcoholic fatty liver disease [22, 23, 24], IBD [25], type 2 diabetes [26], and inflammatory arthritis [27], with bile acids and their derivatives emerging as potential therapeutic agents. Importantly, accumulating evidence indicates that bile acid metabolism is profoundly altered in PSC patients, with elevated serum levels of conjugated primary bile acids, reduced secondary bile acids, and disrupted bile acid synthesis pathways [28, 29]. Thus, bile acid signaling represents a critical therapeutic target in PSC.

In this study, we established a murine model of experimental sclerosing cholangitis (eSC) induced by the DDC (3,5‐diethoxycarbonyl 1,4‐dihydrocollidine) diet and investigated its impact on susceptibility to dextran sulfate sodium (DSS)‐induced colitis. Our findings revealed that dysregulation of bile acid metabolism led to an imbalance of gut immune homeostasis in eSC mice, characterized by decreased CD4^+^ cell counts and a significant increase in the proportion of Th17 cells. Therapeutic administration of LCA and its derivative 3‐O‐LCA ameliorates both hepatic and intestinal pathology via modulation of FXR signaling and immune cell polarization. Our findings provide mechanistic insights into the gut–liver axis in PSC–IBD and support the development of novel bile acid‐based therapeutic strategies for this challenging disease.

## 2 Results

### 2.1 The eSC Mice Exhibit a Markedly Increased Susceptibility to IBD

**FIGURE 1:**
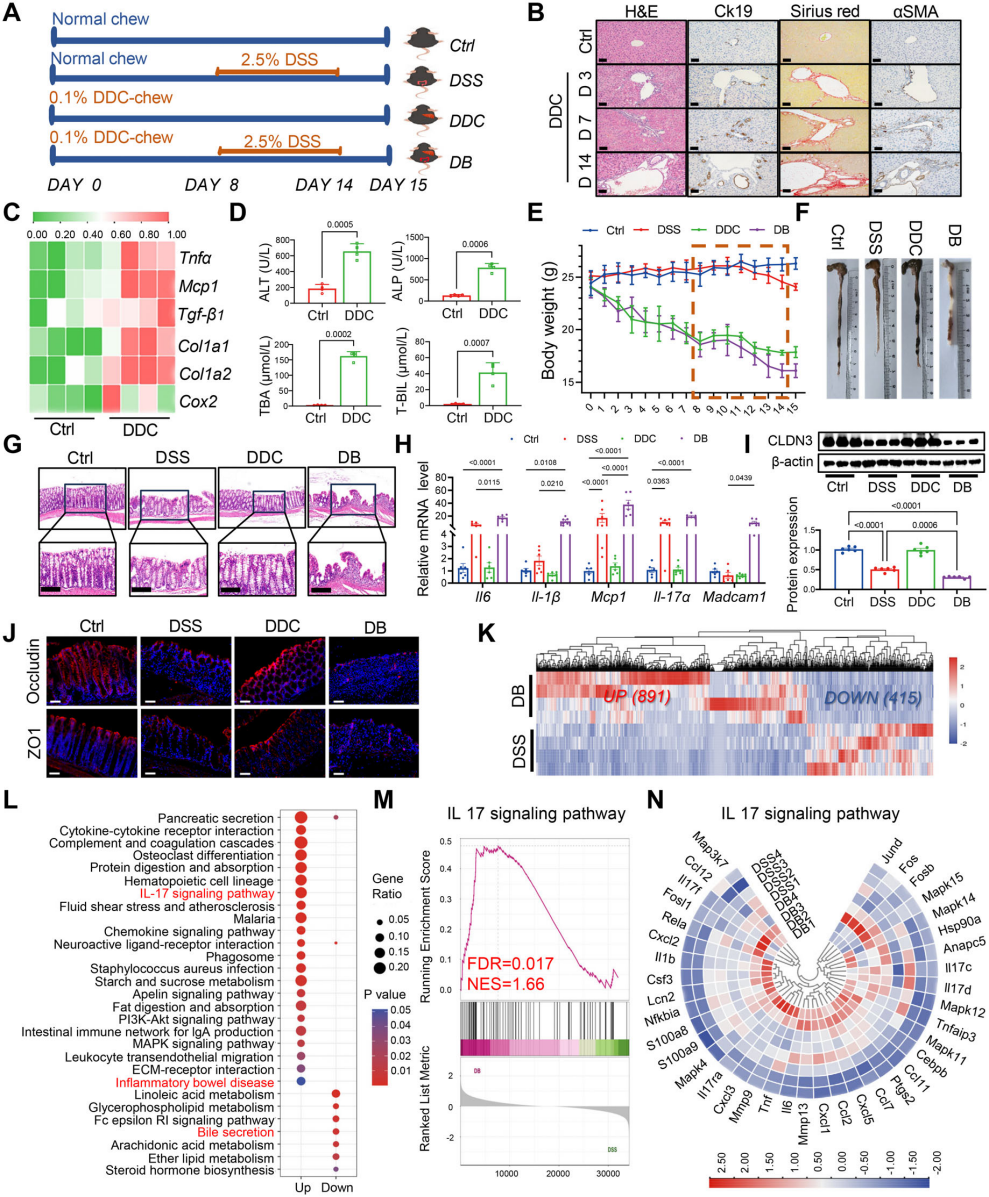
eSC mice exhibited a significantly higher susceptibility to IBD. (A) Schematic illustration of mouse models. (B) Liver tissue sections from mice treated with DDC for varying durations were subjected to staining using H&E, CK19, Sirius red, and anti‐α‐SMA. Scale bars: 50 µm. (C) The heatmap of qPCR results reveals a higher level of hepatic inflammation in mice treated with DDC (*n* = 4). (D) The T‐BIL, TBA, ALT, and ALP levels in the serum were quantified using colorimetry at the end of the DDC treatment (*n* = 4). (E) Alterations in body weight of mice across the four groups. (F) The full‐length colons were isolated from the mice on day 15 and subjected to quantification. (G) Representative images depicting histological sections stained with H&E from colonic tissues of each group. Scale bars: 100 µm. (H) The mRNA level of proinflammatory genes, including *Il6*, *Il‐1β*, *Mcp1*, *Il17a*, and *Madcam1*, in colon tissues were assessed (*n* = 6). (I) Western blot analysis was performed to detect the expression of Claudin‐3 in the colon, and the band intensity was quantified using ImageJ (*n* = 3). (J) The immunofluorescence images demonstrated the staining patterns of ZO‐1 and Occludin in each group. Remarkably, both proteins exhibited a significant reduction in the colon of the DB group mice compared with the DSS group mice. Scale bars: 50 µm. (K‐N) RNA‐seq technology was employed to compare the transcriptome expression profiles of the colonic tissues between the DSS and DB groups. (K) A heatmap illustrating the expression patterns of 1306 DEGs between two groups was generated (*n* = 4). (L) The enrichment analysis of DEGs revealed the most significantly regulated KEGG pathways. (M, N) GSEA was performed, and the leading‐edge subset involved in the IL‐17 signaling pathway was subsequently generated for visualization (*n* = 4). All data are represented as mean ± SEM. *p*‐values were calculated using one‐way ANOVA.

To gain a deeper insight into the association between PSC and IBD, we examined whether this association could be replicated in a murine model, as illustrated in Figure 1A. The eSC model was established by administering the DDC diet (DDC group) as previously reported [30], which resulted in progressive inflammation and fibrosis of the intrahepatic bile ducts. The DDC‐treated mice exhibited a significantly increased expression of cytokeratin 19 (CK19) by day 3, with a continuous increase observed over time, indicating cholangiocyte hyperplasia (Figure 1B; Figure S1A). Elevated levels of α‐smooth muscle actin (α‐SMA) reflected the activation of hepatic stellate cells and myofibroblasts, signifying the progression of liver fibrosis. Additionally, positive results of Sirius red staining were observed three days after initiating DDC administration. After 14 days, typical lesions of sclerosing cholangitis, including inflammatory cell infiltration, bile duct proliferation, and fibrosis, were observed. The supporting evidence also included significant upregulation of mRNA expression levels of genes related to liver inflammation and fibrosis, such as *Tnfa, Mcp‐1, Tgf‐b1, Cola1, Cola2*, and *Cox2* (Figure 1C). Furthermore, the DDC‐treated mice exhibited an increased level of total bilirubin (TBIL), alanine aminotransferase (ALT), alkaline phosphatase (ALP), and total bile acid (TBA) in mouse serum (Figure 1D). These pathological findings reflect pathological conditions that resemble the characteristic pathology observed in human PSC patients, thereby confirming the successful establishment of our eSC mouse model.

Next, we conducted a comparative analysis to assess the impact of DSS administration on mice fed with a regular diet (DSS group) or the DDC diet (DB group). The weight curve demonstrated a pronounced acceleration in the mice's weight loss in the DB group (Figure 1E), accompanied by soft and bloody stool during the later administration stages. On day 15, humane euthanasia was performed on all groups, and the colons were isolated. A significantly shortened colon length was observed in mice of the DB group (Figure 1F; Figure S1B). Histological examination using H&E staining revealed that the DB group of mice exhibited a more pronounced IBD‐like phenotype, characterized by sloughing of intestinal epithelial cells, destruction of crypt structures, and significant collagen fiber proliferation in the colonic mucosa and submucosa (Figure 1G). We also observed more severe pathological scores in the DB group compared with the DSS group (Figure S1C). Subsequently, real‐time PCR was employed to assess the expression levels of specific inflammatory factors in the colonic tissues. Following DSS treatment, *Il‐1β, Mcp‐1*, and *Il‐17α* mRNA were significantly upregulated. This upward trend was even more pronounced in the DB group, where the expression of *Il‐6* and *Madcam1* mRNA was also significantly increased (all *p *< 0.05, compared with both control and DSS groups) (Figure 1H). In addition, results from WB (Figure 1I) and immunofluorescence staining (Figure 1J; Figure S1D) also indicated more severe disruption of tight junction structures in the DB group, which may lead to more significant infiltration of inflammatory cells and microbes, and subsequently trigger a more robust inflammatory response.

Then, we compared the transcriptional expression profiles of colonic tissues between the DSS and DB model groups using RNA‐seq technology. Principal component analysis (PCA), volcano plots, and heatmaps showed distinct separation between the two groups (Figure 1K; Figure S1E, F). Gene Ontology (GO) and Kyoto Encyclopedia of Genes and Genomes (KEGG) analyses were performed on 1306 differentially expressed genes (DEGs) to further understand their functions. GO analysis indicated that upregulated DEGs in the colonic tissue from DB model mice were mainly involved in immune response, leukocyte chemotaxis, positive regulation of IL‐6 production, etc. In contrast, the down‐regulated DEGs were predominantly involved in defense response, programmed cell death, etc. (Figure S1G). KEGG pathway analysis highlighted significant enrichments in pathways related to bile secretion, inflammatory bowel disease, and IL‐17 signaling, as well as pathways like PI3K‐Akt signaling and MAPK signaling, all critical for inflammation and immune responses (Figure 1L). Of note, the IL‐17 signaling pathway plays a crucial role in the pathogenesis of IBD [31, 32], mainly through stimulating epithelial cells and fibroblasts to produce proinflammatory cytokines and chemokines such as IL‐6, TNF‐α, and CCL12, thereby exacerbating intestinal inflammation (Figure 1M,N). Collectively, we successfully established the eSC mouse model and provided compelling evidence for the heightened susceptibility of eSC mice to IBD.

### 2.2 Dysregulation of Immune Cell Homeostasis in the Colonic Tissue Samples From eSC Mice

**FIGURE 2:**
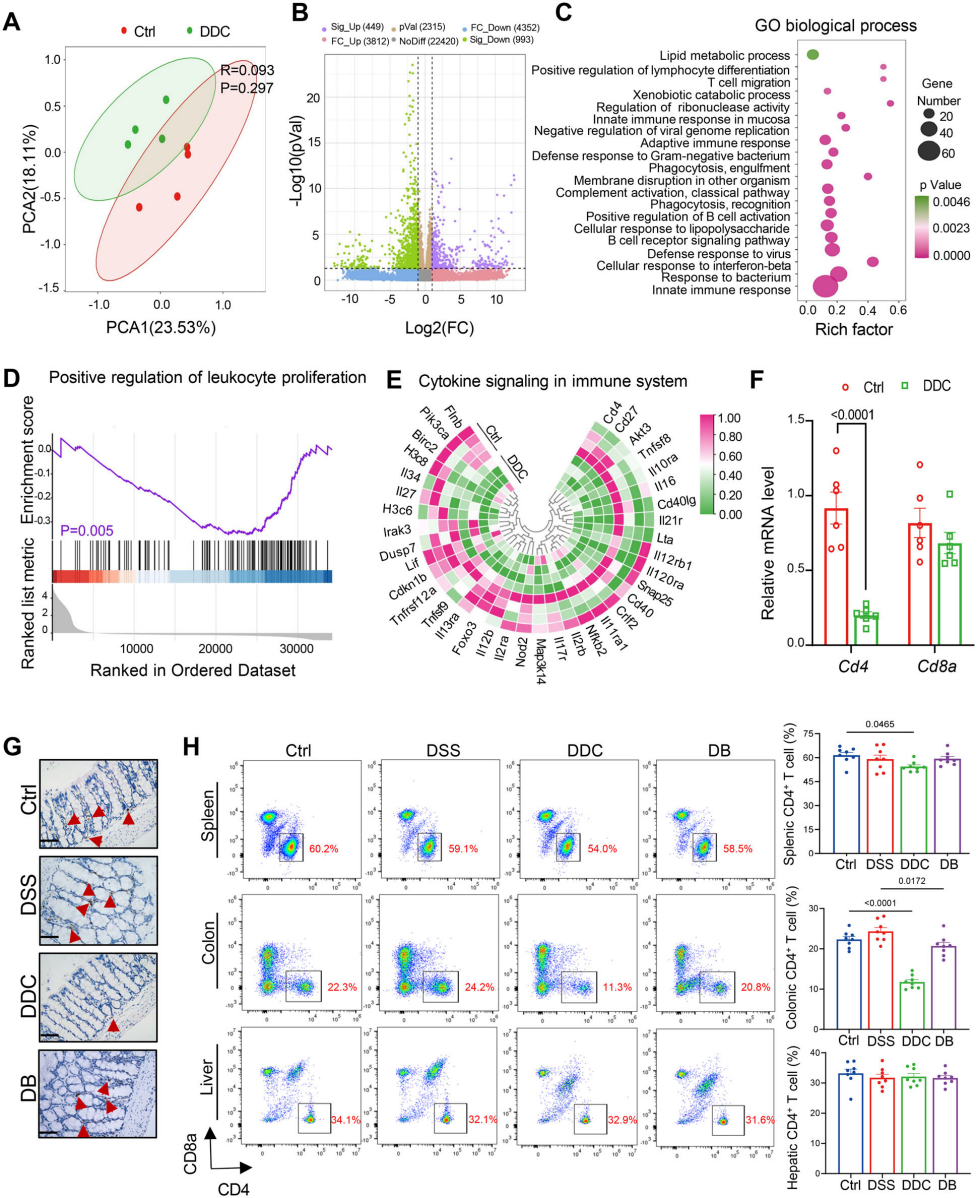
eSC mice demonstrated a specific reduction of CD4^+^ T lymphocytes in the colon. (A) The PCA plot revealed a distinct separation between the two groups, indicating significant differences in sample characteristics (*n* = 4). (B) The volcano plot revealed a total of 1442 DEGs (*p* < 0.05 and |log_2_FC|>1) in the colon tissues of eSC mice. (C) GO analysis revealed the most significantly regulated GO annotations of the DEGs. (D) GSEA analysis indicated a significant downregulation of the ‘positive regulation of leukocyte proliferation’ in eSC mice. (E) A circular heatmap illustrating 37 downregulated genes involved in “cytokine signaling in the immune system” in eSC mice compared with the control group (*n* = 4). (F) The expression level of *Cd4* in the colon was reduced by the DDC diet, while no significant change was observed in *Cd8* expression (*n* = 6). (G) Representative immunohistochemical images depicting the expression of CD4 protein across the groups were obtained. Scale bars: 50 µm. (H) Representative FACS plots of CD4^+^ T cells in the spleen, colon, and liver tissues were generated, with cells gated on CD3^+^ T cell subpopulations (*n* = 8). All data are represented as mean ± SEM. *p*‐values were calculated using unpaired, two‐tailed *t*‐tests.

To investigate the underlying mechanisms contributing to the increased IBD susceptibility in eSC mice, we employed RNA‐seq to compare gene expression profiles of colonic tissues from DDC‐treated mice and healthy controls (Figure 2A). Overall, we identified 449 upregulated and 993 downregulated genes (Figure 2B). GO analysis indicated that these DEGs are involved in processes such as positive regulation of lymphocyte differentiation, T cell migration, innate immune response, and adaptive immune response (Figure 2C). These findings are consistent with the results of Gene Set Enrichment Analysis (GSEA) (Figure 2D), suggesting altered immune cell homeostasis in DDC‐treated mice. KEGG analysis revealed significant enrichment of immune‐related pathways, particularly with 37 downregulated DEGs enriched in the cytokine signaling within the immune system pathway (Figure 2E). qPCR was performed to validate the specific expression of DEGs (Figure 2F). These results confirmed specific expression changes in DEGs, highlighting a significant decrease in *Cd4* gene expression in the colonic tissue of DDC‐treated mice, corroborated by immunohistochemical staining (Figure 2G). In contrast, CD8 expression remained stable, indicating that CD8^+^ T cells were less affected. This observation suggests a specific influence of DDC‐induced cholangitis on the intestinal immune environment. Moreover, a significant reduction in the proportion of CD4^+^CD8^−^ T cells, a population critical for immune regulation, was observed in spleen and colonic tissues isolated from eSC mice, while the proportion remained unchanged in liver tissue (Figure 2H). Overall, DDC‐induced cholangitis appears to substantially impact the gut microenvironment, potentially involving complex processes such as apoptosis, differentiation, and migration.

**FIGURE 3:**
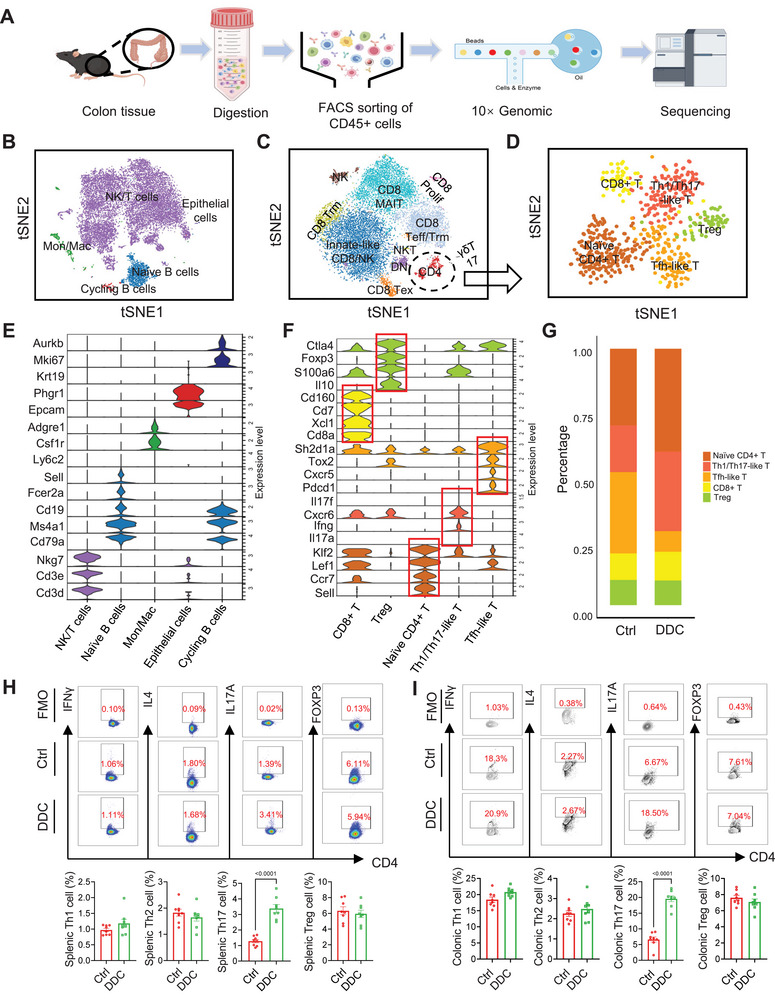
Single‐cell profiling of colonic immune cells revealed an expansion of naïve CD4^+^ T cells and enhanced Th17 differentiation in eSC mice. (A) A schematic representation of the experimental workflow for scRNA‐seq analysis was provided. (B) A t‐SNE plot was generated depicting the clustering of CD45^+^ immune cells isolated from colon tissues. Different cell types were distinguished by different colors (Ctrl, *n* = 9143 cells; DDC, *n* = 7079 cells). (C) A violin plot illustrated the average expression levels of specific lineage‐based marker genes. (D) Subgroup analysis was conducted on NK/T cells from Figure 3B. (E) The *t*‐SNE plot revealed the result of reclustering of CD4 cells from (D). (F) The violin plot displayed the expression of marker genes used to define the cluster functional annotations. (G) Cluster composition per condition was assessed in both the Ctrl and DDC groups. (H, I) Mice were sacrificed on day 15 of DDC treatment, and their spleen and colon tissues were collected for flow cytometry analysis to assess the frequency of Th1, Th2, Th17, and Treg cells, respectively (*n* = 8). All data are represented as mean ± SEM. *p*‐values were calculated using unpaired, two‐tailed *t*‐tests. rm: resident memory; ex: exhausted; eff: effector; NK: natural killer cell; MAIT: mucosal‐associated invariant T cell; NKT: natural killer T cell; DN: double negative cell; Prolif: proliferating cell.

Subsequently, single‐cell sequencing assays were conducted to further investigate the impact of the “DCC diet” on the intestinal immune cells in mice. As depicted in Figure 3A, fresh colon tissues were first collected from DDC‐treated and control mice, cut into small pieces measuring 2–3 mm, and digested by collagenase IV/DNase I solution. Then, CD45‐positive cells were obtained via FACS and used for single‐cell capture and library preparation using the 10x Genomics Chromium system. The t‐SNE plot revealed that the detected intestinal CD45^+^ cell population primarily comprised NK/T cells, Naïve B cells, cycling B cells, monocytes/macrophages, and a small number of epithelial cells (Figure 3B,C; Figure S2A). Next, we evaluated the NK/T cell subpopulation further based on specific marker genes, resulting in the identification of CD8 Tex, CD8 Trm, CD8 Teff/Trm, CD8 MAIT, innate‐like CD8/NK, NK, CD4, γδT17, DN, CD8 prolif, and NKT (Figure 3D; Figure S2B). Further analysis allowed us to identify naive CD4^+^ T cells, Th1/Th17‐like T cells, Tfh‐like T cells, Treg cells, and CD8^+^ T cells (Figure 3E,F). Relative to the control group, samples from DDC‐treated mice exhibited significant increases in the proportion of naive CD4^+^ T cells and Th1/Th17‐like T cells, while there was a decrease in the proportion of Tfh‐like T cells. No significant alterations were observed in the proportions of CD8^+^ T cells or Treg cells (Figure 3G). A KEGG analysis of DEGs within the Th1/Th17‐like T cell population revealed pathways primarily involving hematopoietic cell lineage and Th17 cell differentiation (Figure S2C). Subsequently, flow cytometric analyses were performed on lymphocytes isolated from two groups of mice. Compared with control mice, the proportion of Th17 cells was significantly elevated in both the spleens and colons of DDC‐treated mice, while no statistically significant differences were observed in the percentages of Th1 cells, Th2 cells, or Treg cells between the two groups (Figure 3H,I). Similar findings were observed between the DB and DSS groups (Figure S2D,E). Of note, recent studies have indicated that under pathological conditions, hyperactivated Th17 cells may promote the development of IBD [33, 34].

Taken together, these findings reveal the profound impact of DDC‐induced experimental sclerosing cholangitis on the intestinal immune microenvironment, especially regarding the selective effects on the T cell subpopulation, and such alterations in immune homeostasis may increase the risk of intestinal infection.

### 2.3 Dysbiosis of Gut Microbiota and Disruption of Bile Acid Metabolism in eSC Mice

**FIGURE 4:**
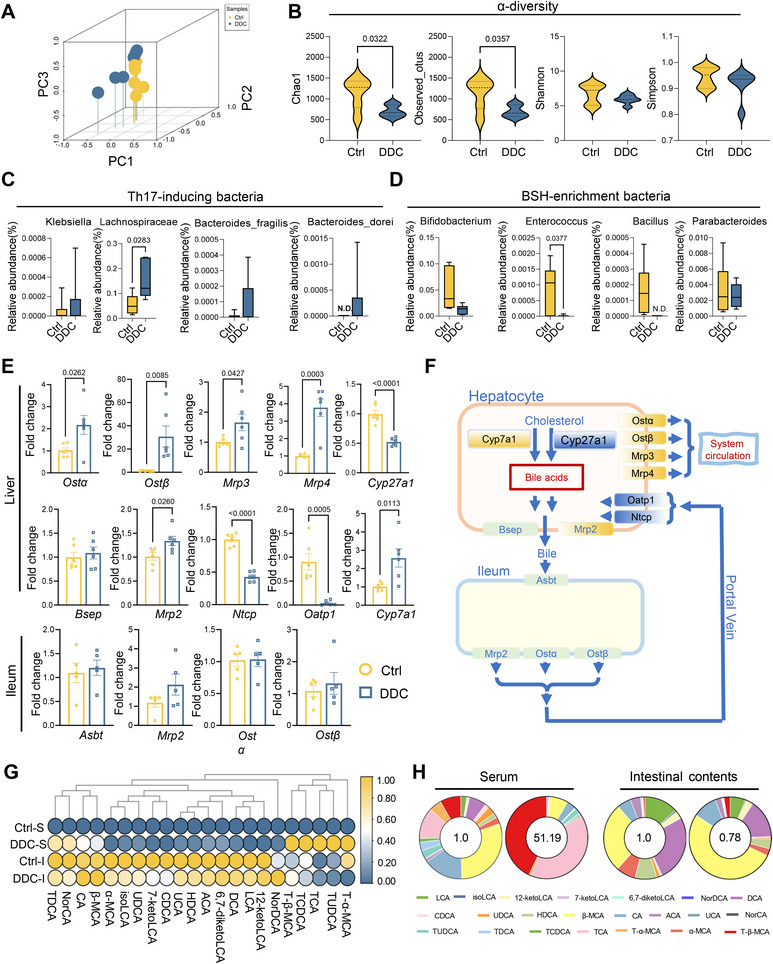
The DDC diet reduced intestinal BSH microbial abundance while simultaneously increasing levels of conjugated bile acids in both intestinal contents and serum. (A) A three‐dimensional PCA diagram discriminated the microbial composition of intestinal contents between the control and DDC groups (*n* = 6). (B) Alpha diversity analysis, including Chao1, Observed_otus, Shannon, and Simpson indices, was conducted on intestinal contents obtained from both groups. (C) Boxplots were used to illustrate the relative abundance of Th17‐inducing bacteria in the intestinal contents for each group. (D) The boxplots demonstrated the relative abundance of BSH‐enrichment bacteria at the genus level. (E) qPCR analysis of genes related to bile acid transport and synthesis in the gut–liver axis reveals a bile acid circulation disruption in eSC mice (*n* = 6). (F) Schematic representation of the findings depicted in (E). (G) Heatmap illustrating bile acid composition in serum or intestinal contents from the two groups. The circles were colored based on standardized expression levels (*n* = 6). (H) The percentage of bile acids isolated from serum or intestinal contents was depicted by pie charts. All data are represented as mean ± SEM. *p*‐values were calculated using unpaired, two‐tailed *t*‐tests.

Mounting research evidence suggests that the intricate interplay among microbiota, bile acid, and immune cells constitutes a sophisticated regulatory network that plays a pivotal role in maintaining homeostasis in the human body [35, 36, 37]. As shown in Figure 4A, three‐dimensional PCA revealed a distinct separation between the gut microbiota profiles of the control and eSC mice. Alpha diversity analysis indicated significant differences in species richness (Chao1) and observed operational taxonomic units (OTUs) between the two groups (all *p* < 0.05; Figure 4B). This included an increase in Th17‐inducing bacteria, such as *Lachnospiraceae* (Figure 4C), which may correlate with the observed increase in the proportion of Th17 cells in the colonic tissue of eSC mice. Moreover, significant alterations in microbes enriched with bile salt hydrolases, such as Enterococcus, have been observed in eSC mice (Figure 4D). The mRNA expression analysis of pivotal genes involved in bile acid circulation was subsequently performed (Figure 4E). A significant upregulation of Cholesterol 7α‐hydroxylase (*Cyp7a1*) expression was observed in the livers of eSC mice, while Cholesterol 27α‐hydroxylase (*Cyp27a1*) showed a significant downregulation. This observation suggests that eSC mice exhibited a higher conversion rate of cholesterol to 7α‐hydroxycholesterol, which represents the initial and pivotal step in bile acid synthesis. Ostα‐Ostβ (organic solute transporter α‐β) is a heterodimeric transport protein located on the basolateral membrane of intestinal epithelial and hepatocyte cells. This transporter is responsible for the absorption of bile acids from the intestine and their secretion into the liver, thereby maintaining enterohepatic circulation. Both genes exhibited significant upregulation in the liver of eSC mice, while no significant changes were observed in the intestines. In general, the hepatic uptake of conjugated bile acids is facilitated by both sodium taurocholate cotransporting polypeptide (Ntcp) and organic anion transporting polypeptides (Oatp), which are respectively expressed on the basolateral and sinusoidal membrane of hepatocytes. Both *Ntcp* and *Oatp* genes were significantly downregulated in the liver of eSC mice. Additionally, there was a notable upregulation in the expression levels of *Mrp2, Mrp3*, and *Mrp4* in the eSC mice liver, potentially serving as a protective response against cholestatic toxicity. Overall, the coordinated action of these genes ensures the proper synthesis, transport, and circulation of bile acids, which is crucial for maintaining bile acid homeostasis in healthy control mice. However, this regulatory balance appeared to be evidently impaired in eSC mice (Figure 4F).

Subsequently, bile acids were quantitatively measured. We observed a significant elevation in TDCA, NorCA, CA, β‐MCA, T‐β‐MCA, TCDCA, TCA, TUDCA, and T‐α‐MCA levels in the serum of eSC mice. Meanwhile, the intestinal contents showed a significant increase in levels of T‐β‐MCA and NorDCA, while there was a decrease in LCA levels (Figure 4G). An overall reduction in the ratio of unconjugated to conjugated bile acids was observed in the intestines of eSC mice (Figure S3A). Further analysis revealed a declining trend in CA/TCA, CDCA/GCDCA, DCA/TCDAC, and β‐MCA/T‐β‐MCA (Figure S3B). We found a significant reduction in serum β‐MCA levels (from 30.13% to 7.19%) in eSC mice, accompanied by a fivefold increase in serum T‐β‐MCA (from 8.15% to 42.97%, Figure 4H; Figure S3C). T‐β‐MCA, a primary bile acid synthesized through hepatic cholesterol metabolism, functions as an endogenous antagonist of FXR, thereby exerting inhibitory effects on FXR activity in the intestinal tract. The aberrant elevation of T‐β‐MCA is consistent with the upregulation of *Cyp27a1* mRNA in the liver, which perturbs bile acid synthesis and metabolism, potentially resulting in dysregulation of bile acid homeostasis. Previous studies have demonstrated that T‐β‐MCA can also indirectly modulate bile acid metabolism by affecting intestinal microbiota composition, including a reduction in *lactobacilli* and other microbial species. Meanwhile, a significant reduction in the proportion of LCA was also observed (from 13.77% to 7.05% in intestinal contents, 2.37% to 0.143% in serum; Figure S3D). LCA, a secondary bile acid, acts as a potent agonist for both FXR and PXR [38, 39], thereby exerting control over de novo bile acid synthesis. Additionally, it can regulate the expression of bile acid transport proteins such as BSEP and NTCP, thereby influencing the enterohepatic circulation of bile acids [40]. Therefore, it is postulated that the dysbiosis of gut microbiota and perturbation in bile acid metabolism observed in eSC mice may be associated with elevated T‐β‐MCA levels and reduced LCA concentrations.

### 2.4 LCA and Its Derivative 3‐O‐LCA Can Effectively Alleviate the Symptoms in eSC and eSC‐IBD Mouse Models by Modulating Immune Response

**FIGURE 5:**
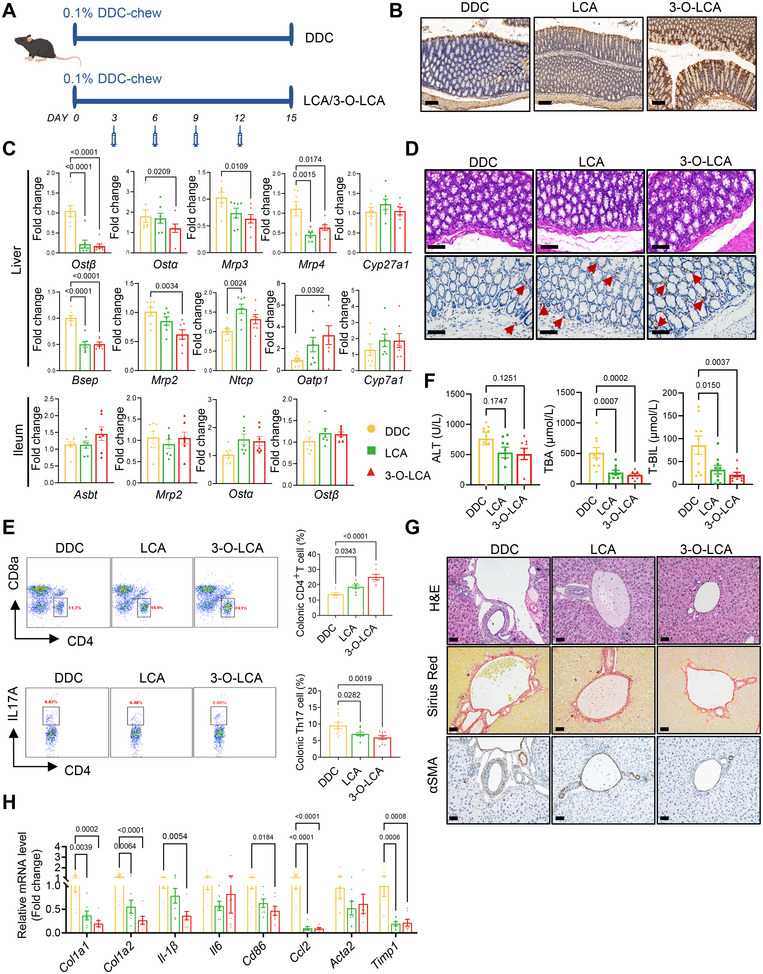
LCA and 3‐O‐LCA administration mitigated intestinal immune imbalance and improved liver function in eSC mice. (A) The experimental design of bile acid administration in mice was conducted as follows: The LCA group received 4 mg of LCA powder dissolved in 200 µL of water via rectal administration on days 3, 6, 9, and 12, similar to the protocol followed by the 3‐O‐LCA group (*n* = 8). (B) Representative images of FXR expression in colon tissues were captured, respectively. Scale bars: 100 µm. (C) qPCR analysis of genes involved in bile acid transport and synthesis in the gut–liver axis revealed improved bile acid circulation following bile acid treatment (*n* = 7). (D) Representative images of colon tissue sections isolated from each group, stained with H&E or anti‐CD4. Scale bars: 100 µm. (E) Fresh colon tissues were collected at the end of treatment and subjected to flow analysis of CD4^+^ (gated on CD3^+^) and Th17 (gated on CD4^+^) cells (CD4: *n* = 8; Th17: *n* = 10). (F) Administration of LCA or 3‐O‐LCA significantly ameliorated serum liver index, including ALT, TBA, and T‐BIL (*n* = 9). (G) Liver tissues from mice exposed to vehicle or bile acids were sectioned and stained with H&E, Sirius red, and anti‐α‐SMA. Scale bars: 50 µm. (H) LCA and 3‐O‐LCA administration led to downregulated expression of proinflammatory cytokines (*n* = 8). All data are represented as mean ± SEM. *p*‐values were calculated using one‐way ANOVA.

To validate our hypothesis, we administered LCA and its derivative 3‐O‐LCA directly to the distal colon of eSC mice (Figure 5A). Following four administrations, notable improvements were observed in bile acid circulation, intestinal immune environment, and liver function in the model mice. First, LCA and 3‐O‐LCA were observed to enhance FXR expression and activity in colonic tissue (Figure 5B; Figure S4A). Subsequently, the regulation of several genes involved in bile acid metabolism was demonstrated (Figure 5C; Figure S4B). Specifically, treatment with LCA/3‐O‐LCA caused a modest upregulation of *Cyp7a1* and *Cyp27a1*, while *Ostα, Ostβ*, and *Bsep* gene expressions were significantly downregulated, which collectively contribute to reduced bile acid synthesis and secretion from the liver. The increased mRNA expressions of *Ntcp* and *Oatp1* may facilitate the transport of bile acids from the bloodstream back into the liver, thereby alleviating cholestasis. Simultaneously, a significant decrease in the expression of *Mrp2, Mrp3*, and *Mrp4* in the liver reflected reduced cholestatic conditions. Second, immunohistochemical analysis demonstrated an elevated number of CD4^+^ T cells in the colonic tissues of LCA/3‐O‐LCA‐treated eSC mice (Figure 5D; Figure S4C). This finding was supported by flow cytometry data, which revealed a significant increase in the proportion of CD4^+^CD8^−^ T cells (gated on CD3^+^) as well as a notable decrease in the percentage of Th17 cells (gated on CD4^+^) within the colonic tissues of LCA/3‐O‐LCA treated group (Figure 5E, all *p* < 0.05). Additionally, qPCR analysis demonstrated a significant upregulation of *CD4* and a downregulation of Th17‐related genes (including *Il‐17a, Il‐23, Rorc*, and *Il‐22*), Th1‐related gene (*Ifn‐γ*) (Figure S4D). Notably, these effects were more pronounced in the group of 3‐O‐LCA treatment. Furthermore, significant reductions in serum levels of ALT, TBIL, TBA, and D‐BIL were observed, which are vital indicators reflecting the degree of cholestasis and liver impairment. These findings indicate that LCA and its derivative 3‐O‐LCA effectively improved liver function in eSC mice (Figure 5F; Figure S4E). LCA/3‐O‐LCA treatment resulted in effective alleviation of collagen deposition and fibrosis in the livers, as demonstrated by the result of Sirius Red staining and α‐SMA immunohistochemistry (Figure 5G; Figure S4F). These effects were further supported by significant downregulation of mRNA expression levels associated with liver inflammation and fibrosis, including genes *Col1a1, Col1a2, IL‐1β, Cd86, Ccl2*, and *Timp1* (Figure 5H).

**FIGURE 6:**
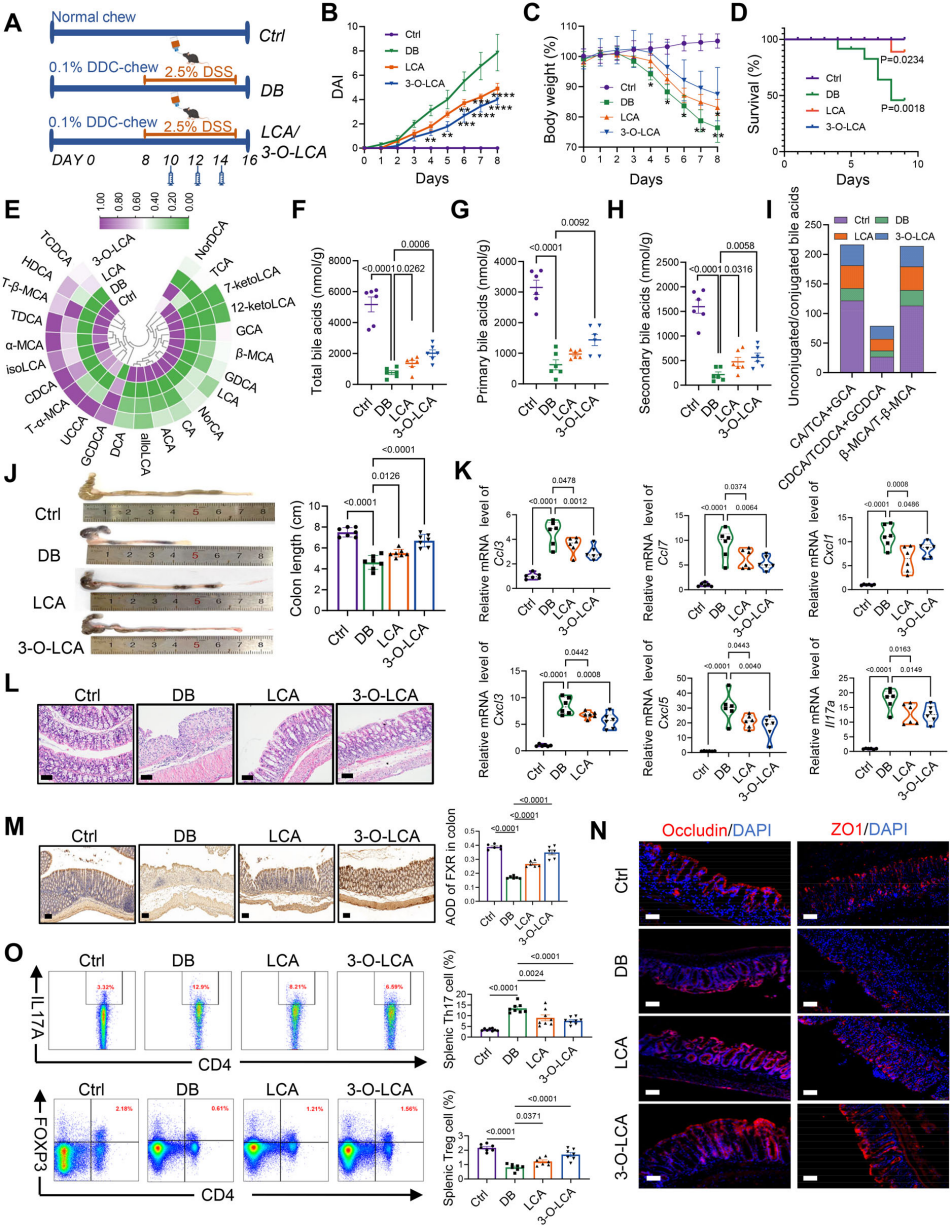
The administration of LCA and 3‐O‐LCA resulted in a reduction in conjugated bile acid levels and ameliorated the severity of colitis in eSC‐IBD mice. (A) Experimental design of bile acid treatment in mice. The LCA group received rectal administration of LCA (8 mg powder dissolved in 200 µL water) on days 10, 12, and 14, as well as the 3‐O‐LCA group (*n* = 7). (B–D) The DAI of colitis, body weight loss, and survival rate was significantly improved following bile acids treatment, respectively. Statistical comparisons between the LCA and DB groups, as well as the 3‐O‐LCA and DB groups, were performed using unpaired two‐tailed *t*‐tests for DAI and body weight data at different time points. Survival curve differences were analyzed using the Log‐rank test. *p < 0.05, **p < 0.01, ***p < 0.001, ****p < 0.0001. (E) Bile Acid Profiling: a heatmap illustrating differentially enriched bile acids in intestinal contents was presented (*n* = 6). (F–H) Bile Acid Classes and Content: Various bile acid classes and their corresponding concentrations were presented (*n* = 6). (I) As an indicator, the impact of LCA and 3‐O‐LCA on BSH activity was evaluated based on the ratio of conjugated to unconjugated bile acids. (J) The full‐length colons were isolated from the mice on day 16 and subsequently quantified. (K) The gene expression of inflammatory cytokines in colon tissues from mice of different groups on day 16 is illustrated using a violin plot (*n* = 6). (L) Representative H&E‐stained histological images in the colons of the eSC‐IBD mouse model following different treatments. Scale bars: 100 µm. (M) The colon tissue sections from mice at the end of treatment were subjected to immunohistochemical staining using anti‐FXR antibodies, respectively. Scale bars: 100 µm. (N) OCT‐embedded colon sections (10 µm) were subjected to immunofluorescence staining for tight junction proteins Occludin and ZO1. Scale bars: 50 µm. (O) Representative FACS images and quantitative analysis revealing the effects of LCA and 3‐O‐LCA on the proportion of Th17 and Treg cells in the spleen (Th17: *n* = 8; Treg: *n* = 7). All data are represented as mean ± SEM. *p*‐values were calculated using one‐way ANOVA.

Subsequently, we investigated whether the administration of LCA and its derivative 3‐O‐LCA can relieve the symptoms of eSC‐IBD in the DB group. These mice were subjected to combined treatment with DDC plus DSS (Figure 6A). As depicted in Figure 6B, administering LCA and 3‐O‐LCA was found to attenuate the disease activity index (DAI) of model mice, indicating an effective mitigation in the severity of inflammatory symptoms. Mice subjected to either intervention exhibited consistently higher body weights than the experimental control group (Figure 6C) and significantly improved survival rates (Figure 6D). Next, absolute quantification of bile acids within the intestinal contents was performed on day 15 of the experiment (Figure 6E). Both treatment groups exhibited significantly increased levels of TBA content (Figure 6F), primary bile acid levels (Figure 6G), and secondary bile acid levels (Figure 6H) compared with the experimental control group, with particularly pronounced differences observed in the 3‐O‐LCA group. Furthermore, both treatment groups showed an elevated unconjugated to conjugated bile acid ratio (Figure 6I; Figure S5A–C). Notably, the colon lengths of mice in the two intervention groups were significantly improved (Figure 6J). The qPCR results revealed varying degrees of downregulation in inflammatory cytokine genes, including *Ccl3, Ccl7, Cxcl1, Cxcl3, Cxcl5*, and *Il17a* in the intestinal samples from both intervention groups, indicating a reduction in inflammation severity (Figure 6K). Moreover, H&E staining confirmed significant improvement in intestinal structure among mice in both intervention groups, characterized by enhanced epithelial microarchitecture, improved crypt architecture, and reduced collagen fiber (Figure 6L). Immunohistochemical results demonstrated enhanced activation of FXR in the colonic tissue of the treatment groups (Figure 6M), potentially contributing to the improved intestinal barrier function, as indicated by upregulated expression levels of tight junction proteins such as Occludin and ZO‐1 (Figure 6N). Furthermore, flow cytometric analysis of splenic lymphocytes revealed a significant decrease in the proportion of Th17 cells and an increase in Treg cells, observed in both treatment groups compared with the experimental control group (Figure 6O). These findings indicate the successful suppression of inflammatory conditions following these interventions. Additionally, a comparative analysis of intestinal microbiota samples among the three groups revealed variations in the relative abundance of specific phyla. However, these differences did not attain statistical significance (Figure S5D), potentially attributed to the relatively short experimental duration. Taken together, these findings demonstrate that both LCA and its derivative 3‐O‐LCA effectively modulate the intestinal immune microenvironment in mice, thereby alleviating the SC‐IBD phenotype.

### 2.5 LCA and 3‐O‐LCA Modulate Macrophage Polarization to Regulate Th17 Differentiation

**FIGURE 7:**
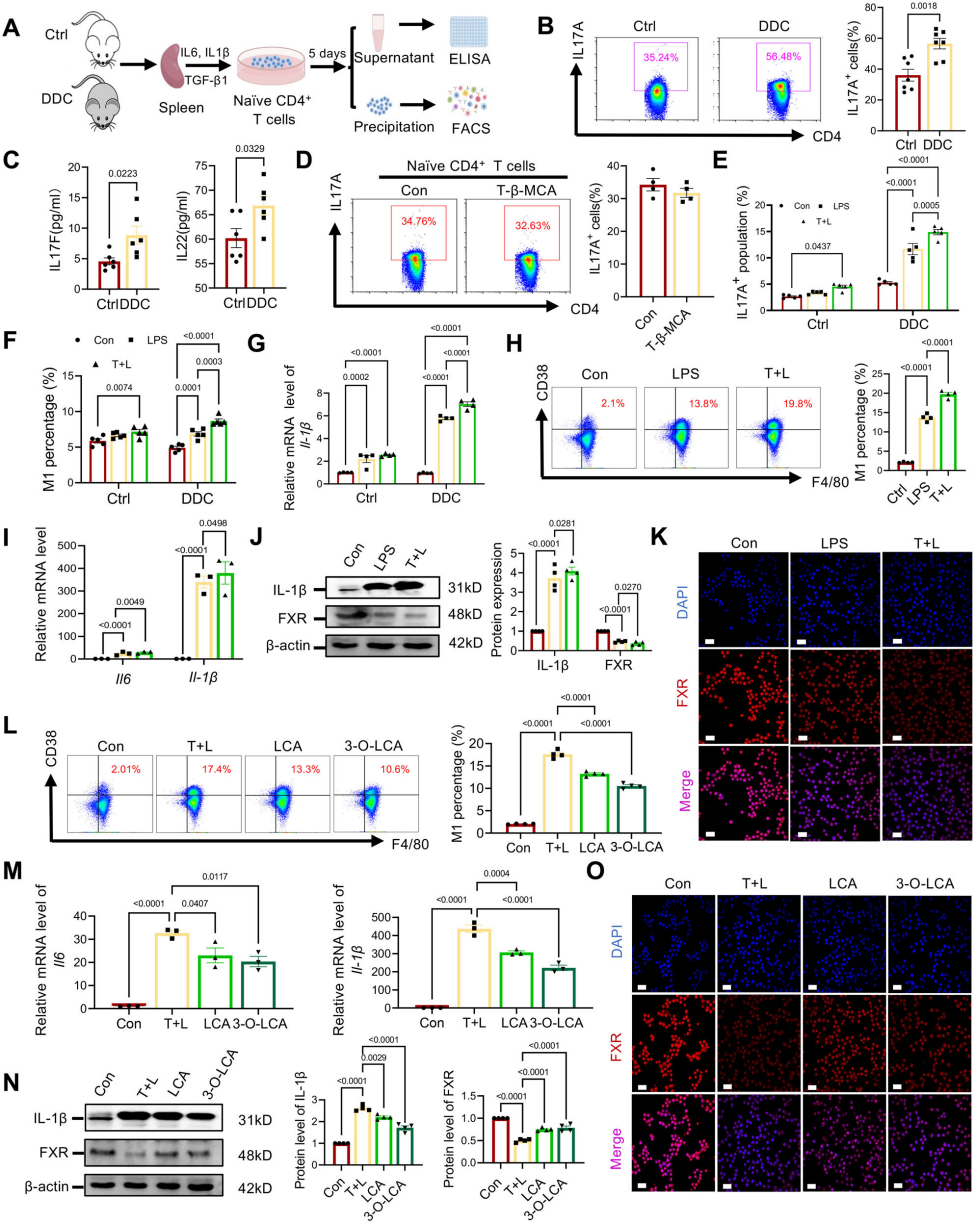
The protective effects of LCA and 3‐O‐LCA are mediated through activating the FXR signal in macrophages. (A) Schematic diagram depicting the experimental design of in vitro Th17 differentiation. (B) The percentage of Th17 was assessed by flow cytometry in purified naïve CD4^+^ T cells cultured for up to 5 days with IL6 (30 ng/mL), IL‐1β (10 ng/mL), and TGF‐β1 (3 ng/mL). (C) Cytokines secreted by Th17 cells in supernatants were quantified using ELISA (*n* = 6). (D) The effect of T‐β‐MCA (25 µM) on spleen‐derived naïve CD4^+^ T cells was evaluated under Th17‐polarizing conditions for 5 days (*n* = 4). (E) Spleen mononuclear cells from control or DDC‐treated mice were pretreated with T‐β‐MCA (25 µM) or DMSO for 3 h, followed by culture in the presence of LPS (100 ng/mL) for 16 h. The Th17 percentage (gated on CD4^+^) was analyzed using FACS (*n* = 5). The “T+L” represents T‐β‐MCA + LPS. (F) The frequency of macrophage M1 polarization of spleen mononuclear cells treated with T‐β‐MCA and LPS was assayed via FACS analysis (*n* = 5). (G) Gene expression level of M1 polarization‐associated *Il‐1β* was determined (*n* = 4). (H) The effect of T‐β‐MCA on M1 polarization of macrophage RAW264.7 was evaluated (*n* = 4). Cells were treated with either vehicle (DMSO) or 25 µM T‐β‐MCA for 1.5 h, followed by stimulation with 300 ng/mL LPS for 6 h. (I) Gene expression of proinflammatory cytokines in RAW264.7 among the three groups (*n* = 3). (J) WB analyses were performed to detect the levels of FXR and IL‐1β in RAW264.7 cells (*n* = 3). (K) Immunofluorescence staining for FXR was conducted on RAW264.7 cells treated with or without T‐β‐MCA or LPS. (L) Representative FACS images demonstrated the impact of LCA and 3‐O‐LCA on M1 polarization of RAW264.7 macrophages (*n* = 4). (M) The mRNA levels of M1 polarization‐associated cytokines were assessed in RAW264.7 treated with or without bile acids (*n* = 3). (N) WB analysis and quantification were performed to determine the FXR and IL‐1β protein levels across the four groups (*n* = 3). (O) Immunofluorescence staining using anti‐FXR antibody was conducted on RAW264.7 cells treated with vehicle or bile acids. All data are represented as mean ± SEM. *p*‐values were calculated using one‐way ANOVA.

Subsequently, we aim to investigate the modulatory effects of LCA and 3‐O‐LCA on these specific subsets of immune cells in mice. First, CD4^+^ naive T cells were isolated from the spleens of the DDC‐treated and healthy control mice using magnetic bead sorting, followed by induction for Th17 differentiation for 5 days (Figure 7A). Flow analysis results revealed a significantly higher proportion of Th17 cells in the DDC‐treated group compared with the controls (Figure 7B). Correspondingly, increased levels of IL22 and IL17 expression in the supernatant were observed via ELISA assay (Figure 7C). Based on our findings and existing literature, we proposed that the significantly elevated levels of T‐β‐MCA in the serum of eSC mice may be implicated in this alteration of fate determination of CD4^+^ naive T cells. However, the expected effect was not observed when CD4^+^ naive T cells from healthy control mice were stimulated with a Th17 induction medium supplemented with T‐β‐MCA. Specifically, there was no significant alteration in the Th17 differentiation ratio (Figure 7D). This finding suggests that the impact of T‐β‐MCA may be attributed to an indirect mechanism. Then, we expanded the initial cell population of Th17 differentiation to mononuclear cells, which were preincubated with 25 µM T‐β‐MCA for 3 h. The monocytes were then exposed to 100 ng/mL LPS for another 16 h. The results demonstrated that pretreatment with T‐β‐MCA significantly enhanced Th17 differentiation in both groups, with a more pronounced effect observed in cells from eSC mice (Figure 7E). Additionally, flow cytometry and qPCR analysis revealed a significant increase in the M1 polarization of macrophages within the mononuclear cell populations of both groups (Figure 7F,G), which has been reported to facilitate the differentiation and activation of Th17 cells.

Next, we conducted in vitro experiments using the RAW264.7 cell line to validate the impact of T‐β‐MCA on macrophage polarization. Notably, T‐β‐MCA significantly increased the proportion of M1 polarization cells in the presence of LPS (Figure 7H; Figure S6A) [41, 42]. We further investigated T‐β‐MCA's potential stimulatory effect on proinflammatory macrophage polarization employing bone marrow‐derived macrophages (BMDMs). As expected, T‐β‐MCA significantly enhanced CD38 fluorescence intensity in BMDMs, aligning with RAW264.7 macrophage responses (Figure S6B). T‐β‐MCA stimulation also resulted in significantly increased mRNA levels of *Il‐6* and *Il‐1β* (Figure 7I) as well as an elevation in protein expression of the potent proinflammatory cytokine IL‐1β (Figure 7J). Concurrently, a decrease was observed in FXR protein expression along with its classical downstream gene small heterodimer partner (*Shp*) (Figure 7J,K; Figure S6C), potentially leading to a more proinflammatory state in RAW264.7 cells and enhancing their susceptibility to inflammatory stimuli. Then, we investigated the effects of LCA and its derivative 3‐O‐LCA on RAW264.7 cells induced with T‐β‐MCA and LPS. The results demonstrated that both LCA and 3‐O‐LCA effectively suppressed M1 polarization in RAW264.7 cells, as evidenced by a decrease in the proportion of F4/80^+^CD38^+^ cells (Figure 7L), a reduction in F4/80^+^CD86^+^ cells (Figure S6D), and lower mRNA expression levels of *Il‐6* and *Il‐1β* (Figure 7M), along with a decline in IL‐1β protein levels (Figure 7N). This finding was corroborated by flow cytometric analysis of BMDMs, which confirmed a significant decrease in M1 polarization ratio (Figure S6E). Furthermore, they promoted the differentiation toward the CD206^+^ M2 phenotype (Figure S6F,G). WB analysis and immunofluorescence staining further confirmed that treatment with LCA and 3‐O‐LCA restored the expression of FXR, along with its classical downstream gene *Shp*, in RAW264.7 cells (Figure 7N,O). In addition, siRNA‐mediated FXR knockdown demonstrated a diminished effect of LCA and 3‐O‐LCA on regulating M1/M2 polarization in RAW264.7 cells (Figure S6H–J), highlighting the crucial role of FXR expression in macrophages during the “beneficial intervention” mediated by LCA and 3‐O‐LCA in eSC and eSC‐IBD model mice.

## 3 Discussion

In this study, we demonstrated that disruption of bile acid metabolism in the gut–liver axis increases the susceptibility of mice to DSS‐induced IBD. DDC is a potent porphyrinogenic agent and an activator of 5‐aminolaevulinate synthetase [43], which can induce obstruction in small ducts and subsequently lead to cholestasis. The DDC diet‐induced mouse model has been widely utilized as an experimental model for sclerosing cholangitis, effectively replicating critical characteristics of human PSC, including the presence of peribiliary and portal–portal fibrosis, enhanced ductal responses, significantly elevated serum bile acid levels, and transformation of cholangiocytes into an activated state [30, 44, 45]. Mice subjected to DDC+DSS treatment exhibited a more pronounced intestinal injury compared with the group treated with DSS alone. This finding was supported by morphological and histological observation, as well as a significant upregulation of inflammatory gene expression, particularly in *Il‐6* and *Il‐1β* mRNA levels, indicating an exacerbated acute inflammatory response in the colon. Additionally, increases in *Mcp‐1* and *Madcam1* mRNA levels suggested enhanced recruitment of monocytes, lymphocytes, and macrophages to the intestine. Elevated *Il‐17a* expression may further contribute to IBD progression by promoting Th17‐cell‐mediated inflammatory responses. The IL‐17 signaling pathway plays a critical role in the mechanisms underlying IBD by stimulating epithelial cells and fibroblasts to produce proinflammatory cytokines and chemokines, such as IL‐6, TNF‐α, and CXCL8. This exacerbates intestinal inflammation and facilitates neutrophil recruitment and activation, which are crucial steps in the inflammatory processes underlying IBD [46]. Dysregulation of the IL‐17 signaling pathway can also compromise intestinal barrier function [31, 47, 48], leading to increased permeability and exacerbating inflammation.

Our findings indicated an immune cell homeostasis imbalance within the colonic tissues of the DDC‐induced eSC mice. RNA‐seq analysis revealed numerous DEGs enriched in immune system cytokine signaling pathways, encompassing genes associated with cytokine receptors, signal transduction, and transcription factors. This extensive downregulation may suppress local immune responses in the colon, reflecting a compensatory mechanism employed by the body to limit excessive inflammatory responses. These findings underscore the impact of sclerosing cholangitis on the intestinal immune microenvironment and emphasize the significance of the gut–liver axis in PSC pathology. CD4^+^CD8^−^ T cells, often regarded as “regulatory commanders” of the immune system, encompass subsets such as helper T cells (Th cells), regulatory T cells (Treg), and naïve T cells. These cell populations modulate antibody production and cytotoxic responses by recognizing antigens presented by antigen‐presenting cells and secreting various cytokines that effectively modulate the overall immune response. Our study demonstrated a significant reduction in CD4^+^CD8^−^ T cell population within the colons of eSC mice, suggesting an altered local immune balance, while CD8 gene expression remained unchanged. This may indicate that the quantity and function of CD8^+^ T cells remain relatively stable, reflecting the selective impact of DDC‐induced sclerosing cholangitis on the intestinal immune environment. Further analysis revealed a significant increase in the proportions of Th17 cells in the colons of eSC mice compared with healthy controls. Th17 cells, primarily localized in the lamina propria of the gut, secrete various cytokines, including IL‐17A, IL‐17F, IL‐21, and IL‐22. Under physiological conditions, IL‐22 secreted by Th17 cells enhances intestinal epithelial cell barrier function and upregulates antimicrobial peptide expression, thereby fortifying intestinal defense mechanisms and maintaining homeostasis. However, dysregulated activation of Th17 cells may contribute to the pathogenesis of IBD.

DDC‐induced sclerosing cholangitis disrupts bile acid metabolism and influences the gut microbiota composition, thereby modulating bile acid production to generate immunoregulatory metabolites that indirectly influence the distribution, abundance, and function of CD4^+^ T cells. Previous investigations have demonstrated a negative correlation between levels of bile acid metabolites such as 3‐oxoLCA and isoalloLCA with the expression of Th17‐related genes [49], with significantly reduced levels of these bile acids observed in IBD patients [50, 51]. This highlights the complex regulatory network among gut microbiota, bile acids, and immune cells. The increased abundance of Th17‐inducing bacteria, such as *Lachnospiraceae*, may correspond to the observed elevation in Th17 cell proportions within the colonic tissue of eSC mice. T‐β‐MCA, a primary bile acid synthesized from cholesterol by hepatic enzymes (such as CYP7A1 and CYP2C70) [52], was significantly evaluated in eSC mice, which leads to over‐suppression of FXR activity in the gut, thereby affecting bile acid synthesis, transport, and metabolism with potential consequences for bile acid homeostasis. Previous studies have also indicated that T‐β‐MCA indirectly regulates bile acid metabolism by altering gut microbial composition [53, 54]. Our results revealed significant changes in the abundance of bile salt hydrolase‐producing microbes (e.g., *Enterococcus*) in the intestines of eSC mice. Additionally, we observed a reduction of intestinal levels of LCA, which acts as a potent FXR agonist [54]. LCA can suppress the expression of CYP7A1, the critical enzyme involved in bile acid synthesis, through activation of the FXR signaling pathway, thereby modulating endogenous bile acid production and regulating the expression of hepatic transporters BSEP and NTCP, consequently impacting enterohepatic circulation of bile acids [55, 56]. We also showed that supplementation with LCA and 3‐O‐LCA effectively enhanced FXR activation in the intestines of eSC mice, leading to significant suppression of intestinal toxic responses, regulation of inflammatory gene expression, restoration of bile acid metabolic homeostasis, inhibition of M1 macrophage polarization, and induction of M2 macrophage polarization. Flow cytometry analysis showed that Th17 cell proportions returned to baseline levels in the treatment group, accompanied by a significant increase in Treg cells. This could be attributed to anti‐inflammatory factors such as IL‐10 and TGF‐β secretion by M2 macrophages, which promote Treg cell differentiation while inhibiting Th17 cell differentiation [57, 58].

While our study provides valuable insights into the gut–liver axis in PSC–IBD, several limitations should be acknowledged. First, the murine DDC diet‐induced eSC model, despite its widespread use, may not fully capture the complexity and heterogeneity of human PSC and its association with IBD. Differences in bile acid profiles and immune system architecture between mice and humans may affect translatability. Second, the relatively short duration of bile acid interventions limited our ability to assess long‐term efficacy and safety. Third, the significant interindividual variability in gut microbiota composition and bile acid metabolism observed in human populations cannot be adequately represented in inbred mice maintained under controlled conditions. Lastly, clinical validation of our findings in PSC–IBD patients is necessary to confirm the therapeutic potential of targeting bile acid signaling pathways. Future research should include extended treatment regimens, a broader range of animal models, and human cohort studies to address these gaps.

In summary, our work highlights the critical interplay among bile acid metabolism, gut microbiota, and immune regulation in PSC–IBD pathogenesis. Targeting bile acid signaling, particularly through FXR modulation by bile acid derivatives such as LCA and 3‐O‐LCA, represents a promising therapeutic avenue. Understanding microbial contributions, especially the role of *Lachnospiraceae* and related taxa, may further support the development of targeted interventions to restore immune homeostasis and prevent disease progression.

## 4 Methods

### 4.1 Mice

Male C57BL/6 mice, aged 8–10 weeks, were purchased from Shanghai SLAC Laboratory Animal Co. The mice were housed under SPF conditions with free access to water and food. All animal procedures were performed in accordance with the guidelines of the Institutional Animal Care and Use Committee at Zhejiang University (application ID: 18021).

### 4.2 Macrophage Culture and Polarization

RAW264.7 cell line was cultured in Dulbecco's modified Eagle's medium (DMEM) with 10% FBS and 1% penicillin/streptomycin in an incubator set at 37°C and 5% CO_2_. For M1 polarization, the cells were pretreated with LCA (TCI, 20 µM), 3‐oxo‐LCA (MCE, 20 µM), or T‐β‐MCA (MCE, 20 µM) for 1.5 h before adding LPS (Sigma, 300 ng/mL) to the culture medium. For M2 polarization, cells were treated with bile acids and subsequently exposed to IL4 (214‐14, PeproTech; 10 ng/mL) for 24 h. After treatment, the cell precipitates were collected by centrifugation and prepared for FACS analysis or RNA extraction.

### 4.3 Flow Cytometry Analyses

Flow assays were performed on a CytoFLEX S (Beckman) flow cytometer, and the data were analyzed by FlowJo 10 (BD Biosciences). For intracellular staining, cells were fixed and permeabilized using the intracellular Fixation & Permeabilization Buffer Set (424402, Biolegend). For nuclear factor staining, cells were fixed and permeabilized using the Foxp3/Transcription Factor Staining Buffer Set (88‐8824‐00, eBioscience). The following antibodies were used to stain the cells: CD4 (100516, Biolegend), CD3 (E‐AB‐F1013D, Elabscience), CD8a (100705, Biolegend), IL17A (506904, Biolegend), IL4 (504103, Biolegend), FOXP3 (E‐AB‐1238D, Elabscience), F4/80 (E‐ab‐F0995D, Elabscience), CD86 (159215, Biolegend), CD38 (102717, Biolegend), and CD206 (141707, Biolegend).

### 4.4 Western Blot Assays

The western blot was conducted following previously established protocols [59]. Briefly, lysates were boiled and separated on 10% SDS‐PAGE gels, followed by protein transfer onto PVDF membranes for subsequent immunoblotting using primary antibodies. After incubation with secondary antibodies, signal detection was achieved using an ECL system (CWBIO), and quantification was performed using ImageJ software. The primary antibodies employed in this study included anti‐FXR (72105, Cell Signaling Technology), anti‐Claudin3 (A2946, Abclonal), anti‐Il‐1β (26048‐1‐AP, Proteintech), anti‐β‐actin (14395‐1‐AP, Proteintech), and anti‐TGR5 (ab72608, Abcam).

### 4.5 Immunofluorescence and Immunohistochemistry Assays

Mice were sacrificed, and the colons were fixed in 4% PFA at 4°C overnight. The samples were then dehydrated, infiltrated, embedded in OCT compound, and sliced into 10 µm‐thick sections using a pathologic microtome (RM2016, Leica). EDTA (PH = 8.0) was used for antigen retrieval, while nonspecific binding was blocked with 3% BSA. The slides were then incubated with primary antibodies, followed by secondary antibodies. Spontaneous fluorescence quenching reagent was added before coverslipping with an anti‐fade mounting medium. Finally, the slides were observed under laser scanning confocal fluorescence microscopy (FV3000, Olympus). The primary antibodies used for immunofluorescence assay included anti‐ZO1(21773‐1‐AP, Proteintech), anti‐Occludin (27260‐1‐AP, Proteintech), and anti‐FXR (25055‐1‐AP, Proteintech).

For the immunohistochemistry study, the colons were isolated and fixed in 4% PFA at room temperature for 48 h and then embedded in paraffin and sliced into 4 µm thick sections by a pathologic microtome (RM2016, Leica). EDTA (PH = 9.0) was used for antigen retrieval, and 3% hydrogen peroxide was used to block endogenous peroxidase. Following the slight drying of the sections, serum sealing was achieved by adding 3% BSA. The sections were then sequentially incubated with primary and secondary antibodies and visualized using DAB staining. Hematoxylin counterstain was applied to visualize nuclei (blue). Primary antibodies used in the immunohistochemistry assay included anti‐CK19 (10712‐1‐AP, Proteintech), anti‐α‐SMA (14395‐1‐AP, Proteintech), and anti‐FXR (25055‐1‐AP, Proteintech).

### 4.6 Statistical Analysis

All data are presented as mean ± standard error of the mean from at least three independent assays. Statistical analysis between groups was performed using the unpaired, two‐tailed Student's *t*‐test (or ANOVA) integrated within the GraphPad Prism software.

## Author Contributions

Ye Chen and Hui Chang conceptualized the study. Hui Chang, Yang Jiang, Qiong Zhao, Zhen Su, Mingyang Chen, Qiufen He, Jingbo Lai, Jing Zheng, and Ruolang Pan performed the experiments and analyzed the data. Yingru Jiang analyzed and compiled the single‐cell sequencing data. Hui Chang, Jian‐Zhong Shao, Robert Chunhua Zhao, and Ye Chen interpreted the data and wrote the manuscript. All authors have read and approved the article.

## Conflicts of Interest

The authors declare no conflicts of interest.

## Ethics Statement

All animal procedures were approved by the Institutional Animal Care and Use Committee at Zhejiang University (approval number: 18021).

## Supporting information




**Figure S1. DB mice exhibited more severe colitis symptoms compared with the DSS group**. (A) Quantification of stained area in Figure 1B, six fields were randomly selected. (B) Quantification of colon length in Figure 1F (*n* = 6). (C) Histopathology scores of Ctrl and DDC‐induced mice, with or without DSS exposure for 7 days (*n* = 6). (D) Quantitative immunofluorescence analysis revealed reduced expression of ZO‐1 and Occludin in colonic tissues from the DB and DSS groups compared with healthy controls, with the DB group exhibiting the lowest expression levels (*n* = 8). (E) PCA plot of RNA‐seq data is presented, demonstrating the principal component analysis results (*n* = 4). (F) Volcano plot illustrated the DEGs, which were identified based on set thresholds (fold change threshold, |log2FC|>1; *p*‐value threshold, *p* < 0.05). (G) Enrichment analysis of DEGs revealed the most significantly regulated GO annotations. All data are represented as mean ± SEM*. p*‐values were calculated using one‐way ANOVA.

## Data Availability

All data generated or analyzed during this study are available from the corresponding authors upon reasonable request. RNA‐seq data have been submitted to the National Center for Biotechnology Information Sequence Read Archive under the accession number PRJNA1152140, and scRNA‐seq data have been submitted to the National Center for Biotechnology Information Sequence Read Archive under the accession number PRJNA1150915.
