# Supplementary material for: Disruption of Bile Acid Metabolism in the Gut–Liver Axis Predisposes Mice to Inflammatory Bowel Disease
Source: MedComm (2020). 2025 Oct 9;6(10):e70429. doi: 10.1002/mco2.70429 (PMC12508617; doi:10.1002/mco2.70429)
Supplement: Supplementary file 1 — Figure S1. DB mice exhibited more severe colitis symptoms compared with the DSS group. (A) Quantification of stained area in Figure 1B, six fields were randomly selected. (B) Quantification of colon length in Figure 1F (n = 6). (C) Histopathology scores of Ctrl and DDC‐induced mice, with or without DSS exposure for 7 days (n = 6). (D) Quantitative immunofluorescence analysis revealed reduced expression of ZO‐1 and Occludin in colonic tissues from the DB and DSS groups compared with healthy controls, with the DB group exhibiting the lowest expression levels (n = 8). (E) PCA plot of RNA‐seq data is presented, demonstrating the principal component analysis results (n = 4). (F) Volcano plot illustrated the DEGs, which were identified based on set thresholds (fold change threshold, |log2FC|>1; p‐value threshold, p < 0.05). (G) Enrichment analysis of DEGs revealed the most significantly regulated GO annotations. All data are represented as mean ± SEM. p‐values were calculated using one‐way ANOVA. [file MCO2-6-e70429-s001.docx]

Supplementary Materials for

Disruption of bile acid metabolism in the gut-liver axis predisposes mice to inflammatory bowel disease

**Hui Chang, Yang Jiang, Qiong Zhao, Zhen Su,** **Mingyang Chen, Qiufen He, Jingbo Lai, Yingru Jiang, Jing Zheng, Ruolang Pan, Jian-Zhong Shao, Robert Chunhua Zhao, Ye Chen**

**This PDF file includes:**

- Supplementary Materials and Methods;
- Figures S1 to S6;
- Tables S1

**Supplementary Materials and Methods**

CD4^+^ T cell differentiation assays

Naive CD4 ^+^ T cells in the spleen were isolated using EasySep Mouse Naïve CD4+ T cell isolation kit (19765, STEMCELL Technologies). For Th17 cells differentiation, 1 × 10^5^ naïve CD4 T cells were cultured on plates coated with anti-mouse CD3e (553057, BD Biosciences; 2 μg/ml) and anti-mouse CD28 (553294, BD Biosciences; 5 μg/ml) in complete RPMI 1640 media [10% FBS, penicillin (100 U/ml), streptomycin (100 μg/ml)]. Recombinant human transforming growth factor-β1 (TGFβ1) (100-21, PeproTech; 3 ng/ml), recombinant mouse IL-1β (211-11B, Pepro Tech; 10 ng/ml), and recombinant mouse IL-6 (216-16, Pepro Tech; 30 ng/ml) were also added to induce differentiation. On day 5, cells were stimulated with PMA, ionomycin, and brefeldin A for 4 hours before harvest for FACS analysis.

Isolation of colonic lymphocytes

The fresh colon tissues were obtained from mice, longitudinally incised and briefly rinsed, then cut into 1 cm segments. Subsequently, the small tissue fragments were incubated in a shaker at 250 rpm and 37°C for 20 min in a pre-digestion solution composed of 15 ml PBS, 5% FBS, 2.5 mM EDTA, and 1 mM DTT. After incubation, the cell suspension was filtered through a 70 μm cell sieve. The filtrate was collected for the subsequent experiment, while the tissue debris was saved for Lamina Propria Lymphocytes (LPL) preparation. The retained tissue debris underwent an additional incubation step with the digestion solution (RPMI-1640 medium, 5% FBS, 1 mg/ml Collagenase Type IV, and 0.02 mg/ml DNase I) in a shaker at 250 rpm and maintained at 37°C for 25 min. Post-incubation, the digested tissues were filtered through a 70 μm cell sieve and combined with the previous filtrate before proceeding further. The lymphocytes were enriched using a 30% Percoll density gradient and washed twice with PBS. For intracellular or nuclear cytokine detection, cells were stimulated with phorbol 12-myristate 13-acetate (PMA) and ionomycin in the presence of brefeldin A and monensin for 6 hours prior to flow cytometric staining.

Isolation of spleen-derived cells

Spleen lymphocytes were obtained using a physical grinding method. To remove red blood cells, an erythrocyte lysate was employed, and single-cell suspensions were generated by passing the samples through a 40 μm cell sieve followed by centrifugation. Spleen-derived mononuclear cell suspensions were obtained using the Mouse Spleen Mononuclear Cell Isolation Solution Kits (P4880, Solarbio) following the manufacturer's instructions. For intracellular or nuclear cytokine detection, cells were stimulated with phorbol 12-myristate 13-acetate (PMA) and ionomycin in the presence of brefeldin A and monensin for 6 hours prior to flow cytometric staining.

Isolation of hepatic lymphocytes

The liver tissue was excised, minced, and digested in 15 mL HBSS supplemented with 0.01% collagenase IV, 0.001% DNase I, 0.02% BSA, and 1 mM CaCl₂ at 37℃ on a roto-mixer for 30 minutes. After washing twice with cold PBS containing 2% FBS, the cell suspension was filtered through a 70-μm nylon strainer and centrifuged at 800 × g for 5 minutes to collect immune cells. Cell pellets were resuspended in 33% Percoll prepared in HBSS and centrifuged at 800 × g for 25 minutes at 4℃, mononuclear cells were collected from the bottom of the tube and washed twice with PBS.

Isolation and polarization of BMDMs

Bone marrow cells were isolated by flushing the femurs and tibiae with ice-cold PBS containing 5% FBS using a 25-gauge needle and 5-mL syringe. The cell suspension was centrifuged at 300 ×g for 5 min, followed by erythrocyte lysis. After filtration through a 70-μm cell strainer, the pellet was resuspended in DMEM supplemented with 10% FBS, 1% penicillin/streptomycin, and 50 ng/mL recombinant M-CSF (HY-P7085, MCE). Cells were seeded in 12-well plates at a density of 5×10^6^ cells per well and maintained at 37°C with 5% CO₂. On day 4, half of the medium was replaced with fresh differentiation medium. On day 7, mature BMDMs were stimulated for 24 h with either LPS (100 ng/mL) to induce M1 polarization or IL-4 (20 ng/mL) to induce M2 polarization, with or without pretreatment with bile acids (specifically 20 μM for LCA/3-O-LCA, 25 μM for T-β-MCA).

Histopathological scores and DAI of ulcerative colitis

Histopathological scoring of colon tissues was based on the sum of five parameters (0-2 points each): goblet cell depletion, crypt apoptosis, epithelial erosion, lymphocytic infiltration, and crypt distortion. The DAI is determined by evaluating three components (0-4 points each): body weight loss, stool consistency, and fecal bleeding. The final DAI value is calculated as the sum of the scores from each category.

siRNA transfection

The transfection experiment was conducted in a 12-well plate 24 hours after seeding, using a jetPRIME transfection kit (Polyplus) following the manufacturer's instructions. The sequences for FXR siRNA were listed as follows: siFXR-1: CCCGAUGUUCAGUUUCUAUAATT (sense), UUAUAGAAACUGAACAUCGGGTT (antisense); siFXR-2: CACAGAUUUCCUCCUCGUCUUTT (sense), AAGACGAGGAGGAAAUCUGUGTT (antisense); siFXR-3: CCAACAGACCCUCCUG GAUUATT (sense), UAAUCCAGGAGGGUCUGUUGGTT (antisense).

Transcriptomics analyses

Total RNA was extracted using Trizol reagent (15596018, Thermofisher) according to the manufacturer's procedure. The quantity and purity of total RNA were analyzed by Bioanalyzer 2100 and RNA 6000 Nano LabChip Kit (5067-1511, Agilent). The sequencing library was constructed using high-quality RNA samples with an RIN number greater than 7.0. Subsequently, the libraries were pooled and submitted to Illumina Novaseq™ 6000 (LC-Bio Technology CO., Ltd., Hangzhou, China) for paired-end sequencing (PE150) with a read length of 2×150 bp, following the recommended protocol provided by the vendor. Reads containing adapters or low-quality bases were filtered using Cutadapt (https://cutadapt.readthedocs.io/en/stable/, version: cutadapt-1.9). The sequence quality was verified using FastQC (http://www.bioinformatics.babraham.ac.uk/projects/fastqc/, 0.11.9). After that, cleaned paired-end reads were produced. The raw sequence data have been deposited to the NCBI Sequence Read Archive (SRA) datasets with accession number PRJNA1152140.

Single-cell sequencing analyses

To characterize CD45^+^ immune cells from the colon, fresh tissues were collected and cut into 0.5 mm^2^ pieces, followed by gentle mechanical dissociation using Lamina Propria Dissociation Kit (130-097-410, Miltenyi Biotec) according to the manufacturer's instructions. Erythrocyte lysis solution was added to remove erythrocytes. Then, the CD45^+^ cells were harvested from Interepithelial lymphocytes (IEL) and Lamina propria lymphocytes (LPL) using FACS sorting. The single-cell suspension was loaded onto the 10× Chromium chip following the instructions in the 10× Genomics Chromium Single-Cell 3' kit (V3), with an expected capture of 8,000 cells. Subsequently, cDNA amplification and library construction were carried out using standard protocols. Illumina sequencing results were converted to FASTQ format using bcl2fastq software (version 5.0.1). The scRNA-seq data were aligned to a reference genome using CellRanger software, and cellular and individual cellular 3' end transcripts were identified and counted. Then the gene expression matrices for all samples were combined in R (version 4.4.2) and converted to a Seurat object using the Seurat R package (version 5.3.0). To filter out low-quality cells from the scRNA-seq data, cells with fewer than 200 or more than 5,000 detected genes, those exhibiting >10% of unique molecular identifiers (UMIs) originating from mitochondrial transcripts, as well as cells with a total RNA count (nCount_RNA) >20,000, were excluded from downstream analyses. Normalization of raw UMI counts was performed using the NormalizeData function in the Seurat package. Highly variable genes were identified using the FindVariableFeatures function. The ScaleData function was used to scale and center the counts in the dataset. Subsequently, data scaling and centering were carried out using the ScaleData function. Dimensionality reduction was conducted using t-distributed stochastic neighbor embedding (t-SNE) via the RunTSNE function. Cell clustering was achieved through the application of the FindNeighbors and FindClusters functions. Marker genes for each cluster were identified using the FindAllMarkers function. Genes with an adjusted p-value <0.05 were considered significantly differentially expressed. Pathway enrichment analysis of differentially expressed genes was performed using clusterProfiler.

16S rRNA sequencing analyses

The DNA from intestinal contents was extracted using the AllPrep DNA/RNA kit (Qiagen). The V3-V4 region of the prokaryotic small-subunit 16S rRNA gene was amplified using primers 341F (5'-CCTACGGGNGGCWGCAG-3') and 805R (5'-GACTACHVGGGTATCTAATCC-3'). Samples were sequenced on an Illumina NovaSeq platform provided by LC-Bio. Paired-end reads were assigned to respective samples based on their unique barcode and truncated by removing both the barcode and primer sequence.

Bile acids quantification

The bile acids in intestine content were extracted in 400 μL methanol (-20℃). After vigorously vortexing for 60 sec, the sample suspensions were further homogenized for 1 min using a grinder at a frequency of 55 HZ. Subsequently, centrifugation was performed at 12,000 rpm and 4℃ for 10 minutes. An appropriate amount of supernatant was transferred to the LC-MS vial. The LC analysis was conducted on an EXion LC Liquid chromatography (AB SCIEX), and metabolite detection by mass spectrometry was performed using AB6500 Plus (AB SCIEX).

Real-time qPCR analyses

Total RNA was extracted from approximately 50 mg tissue using Trizol reagent (B511311, Sangon Biotech) according to the manufacturer's protocol and quantified using spectrophotometry at 260 nm. Complementary DNA (cDNA) was synthesized using the Evo M-MLV Reverse Transcription Kit (AG11728, Accurate Biology). For qPCR analysis, SYBR Green reagents were utilized on a CFX96 Touch detection system (Bio-Rad). The mRNA levels were calculated using the 2–ΔΔCT method and normalized to β-actin levels. The primer sequences are listed in Supplemental Table 1.

**Supplementary Figures**

**
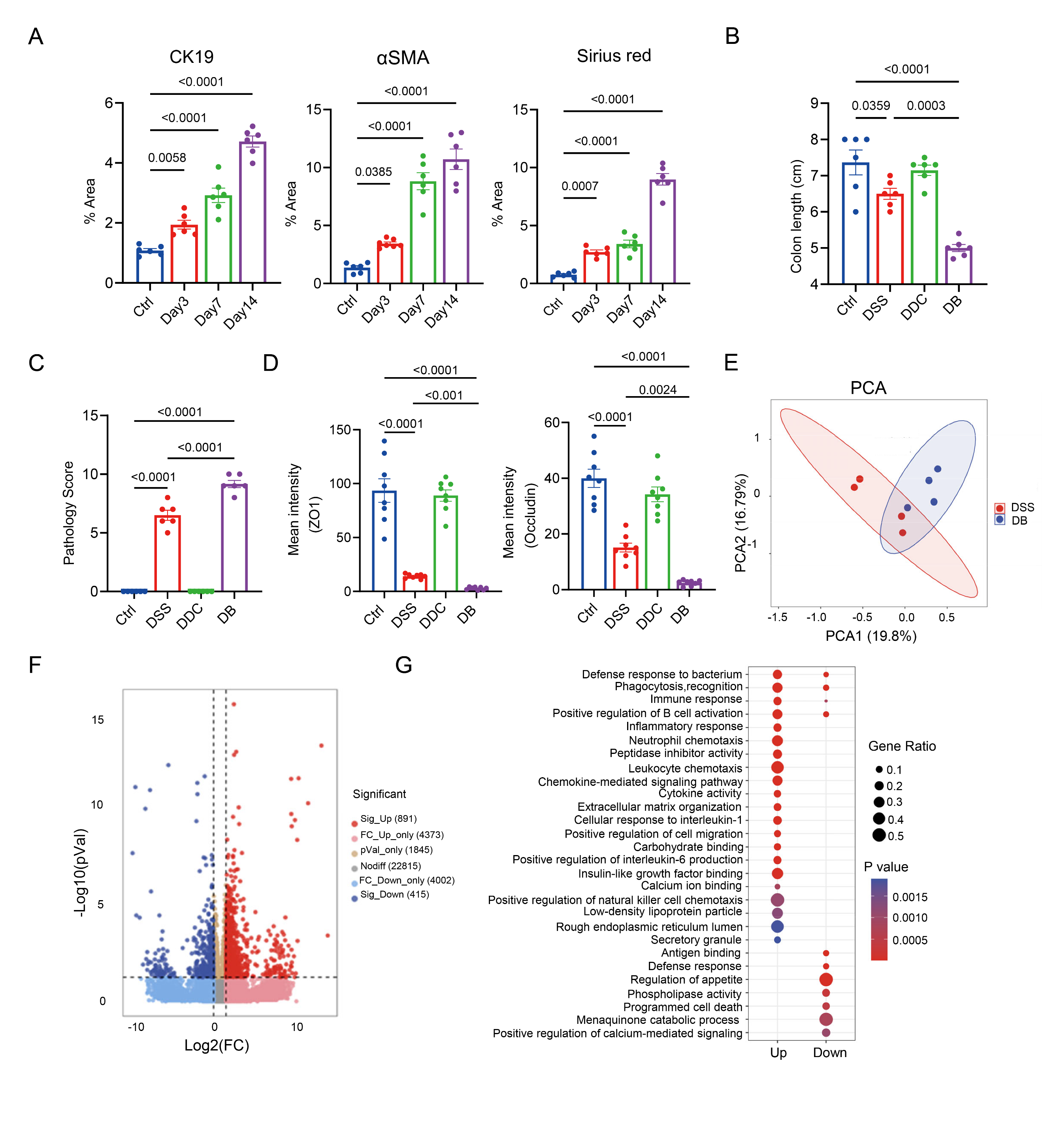
**

**Figure S1. DB mice exhibited more severe colitis symptoms compared to the DSS group.** (A) Quantification of stained area in Figure 1B, six fields were randomly selected. (B) Quantification of colon length in Figure 1F (n=6). (C) Histopathology scores of Ctrl and DDC-induced mice, with or without DSS exposure for 7 days (n=6). (D) Quantitative immunofluorescence analysis revealed reduced expression of ZO-1 and Occludin in colonic tissues from the DB and DSS groups compared to healthy controls, with the DB group exhibiting the lowest expression levels (n=8). (E) PCA plot of RNA-seq data is presented, demonstrating the principal component analysis results (n=4). (F) Volcano plot illustrated the DEGs, which were identified based on set thresholds (fold change threshold, |log2FC|>1; p-value threshold, p<0.05). (G) Enrichment analysis of DEGs revealed the most significantly regulated GO annotations. All data were represented as mean ± SEM*. p* values were calculated using one-way ANOVA.

**
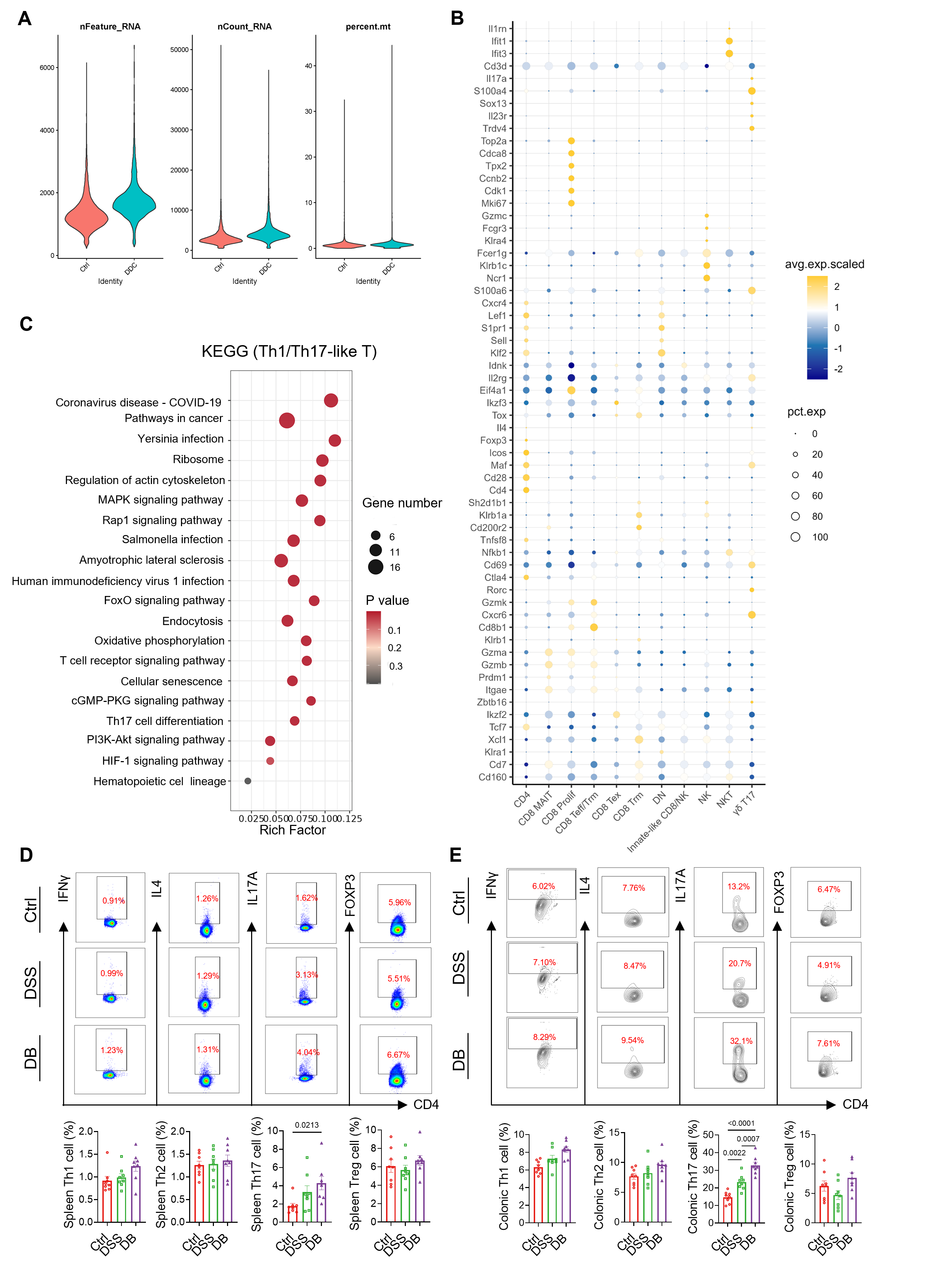
**

**Figure S2. Single-cell atlas of mouse colonic immune cells.** (A) Quality control metrics of the filtered scRNA-seq data used for downstream analysis. (B) Dot plot showing representative genes selected for cluster annotation. (C) KEGG analysis was performed to compare the DEGs in Th1/Th17-likr T cells between the two groups. (D) Representative flow cytometric pseudocolor plots of splenic Th1, Th2, Th17 and Treg cells from three groups (n=8). (E) Representative flow cytometric contour plots of colonic Th1, Th2, Th17 and Treg cells from three groups (n=8).

**
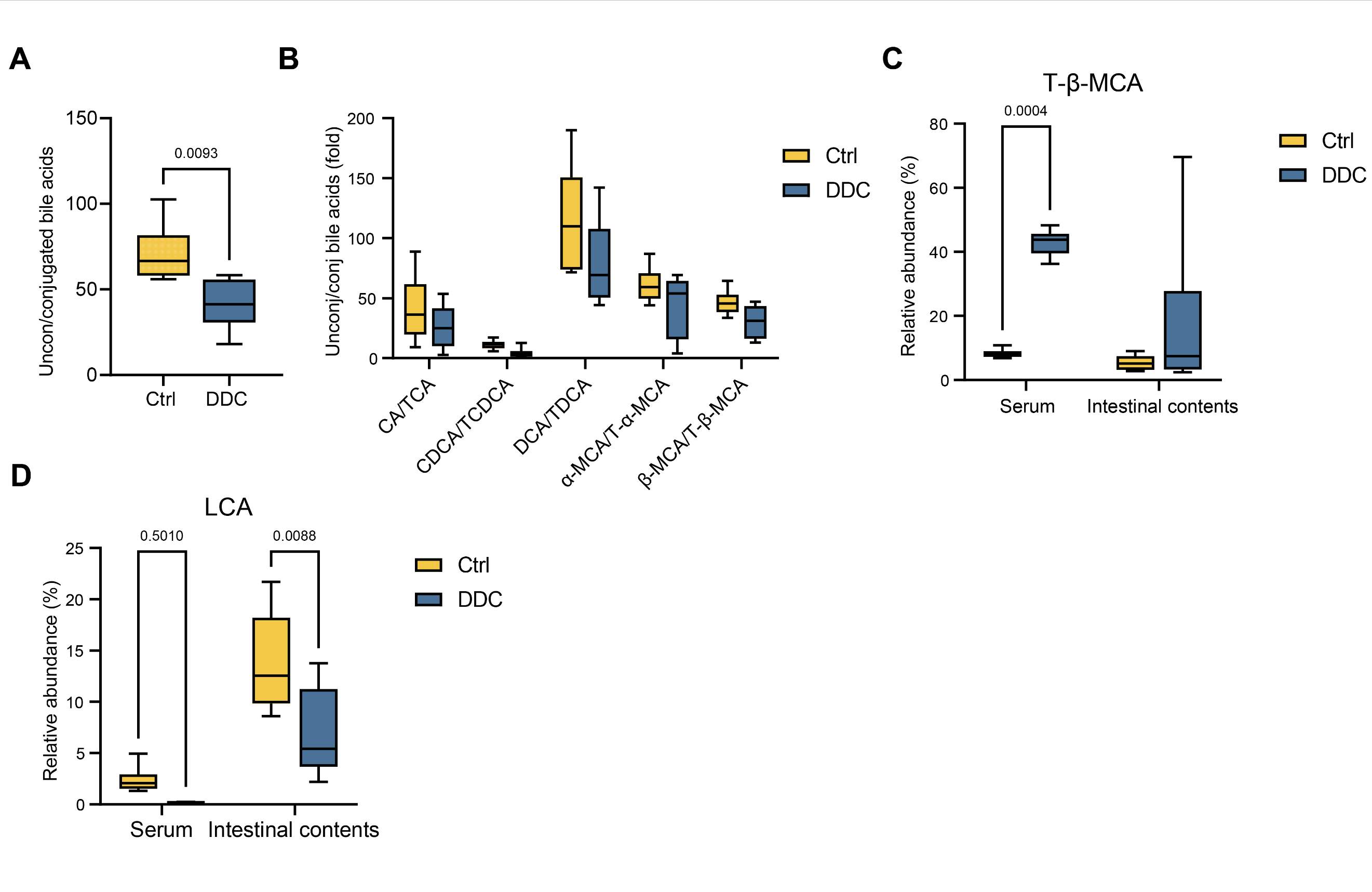
**

**Figure S3. DDC-diet changed the bile acid metabolism in both intestinal contents and serum.** (A) The boxplot discriminated the ratio of conjugated to unconjugated bile acids in intestinal contents. *p* values were calculated using one-way ANOVA. (B) DDC-diet upregulated the ratio of conjugated to unconjugated bile acids in intestinal contents. (C) The relative abundance of T-β-MCA in serum and intestinal contents were presented. *p* values were calculated using two-way ANOVA. (D) Boxplot demonstrating the relative abundance of LCA in serum and intestinal contents. *p* values were calculated using unpaired, two-tailed t-tests.


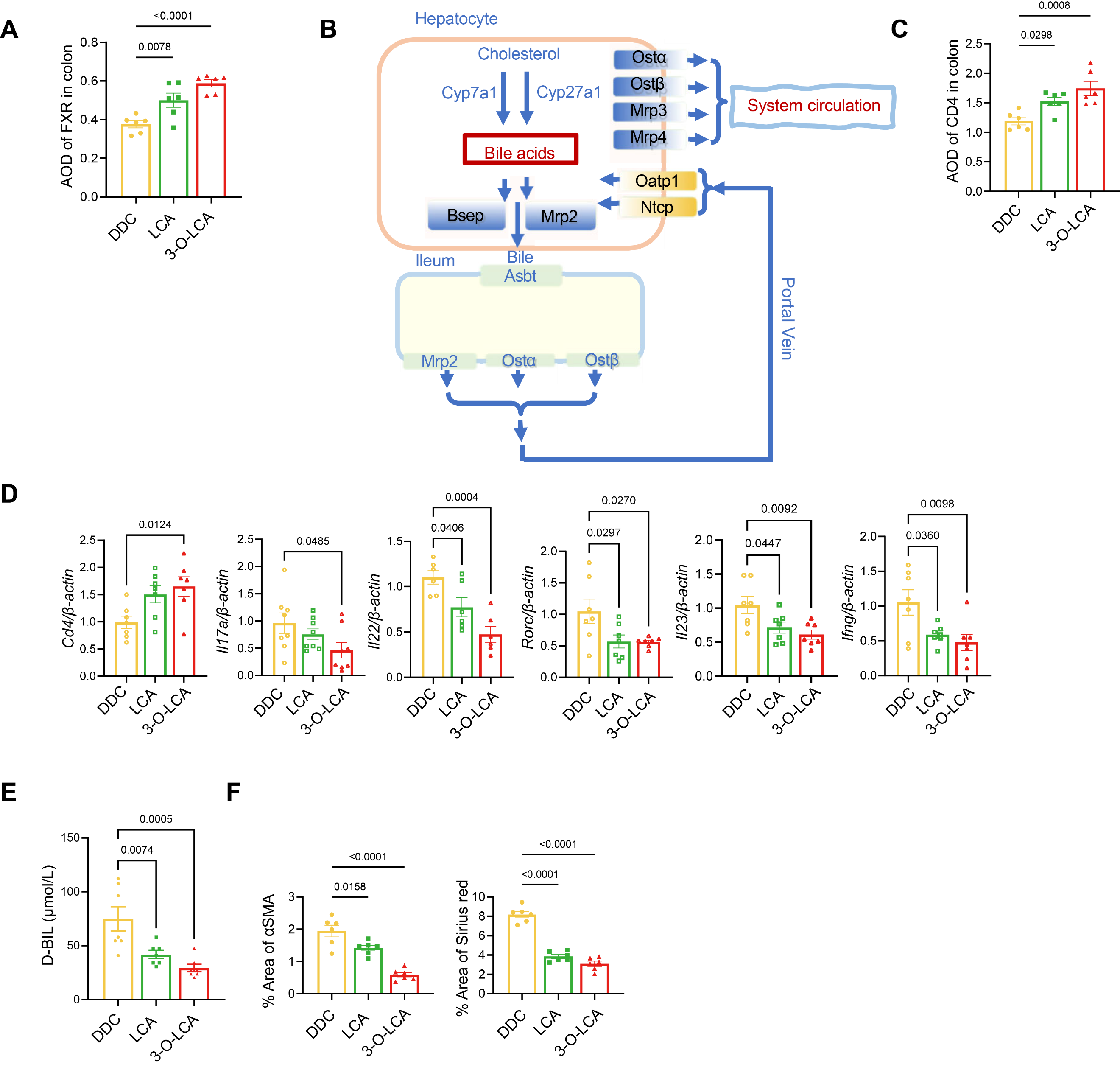


**Figure S4. Effect of LCA and 3-O-LCA on eSC mice model.** (A) Average Optical Density (AOD) analysis of FXR expression based on IHC staining in Figure 5B (n=6). (B) Schematic representation of the findings depicted in Figure 5C. (C) AOD analysis of CD4 expression based on IHC staining in Figure 5D (n=6). (D) qPCR analysis of colonic genes revealed improved *cd4* deficiency and downregulated of genes involved in Th1 and Th17 differentiation following bile acids treatment. (E) Administration of LCA or 3-O-LCA significantly ameliorated serum D-BIL level (n=7). (F) All data were represented as the mean ± SEM, *p* values were calculated using one-way ANOVA.


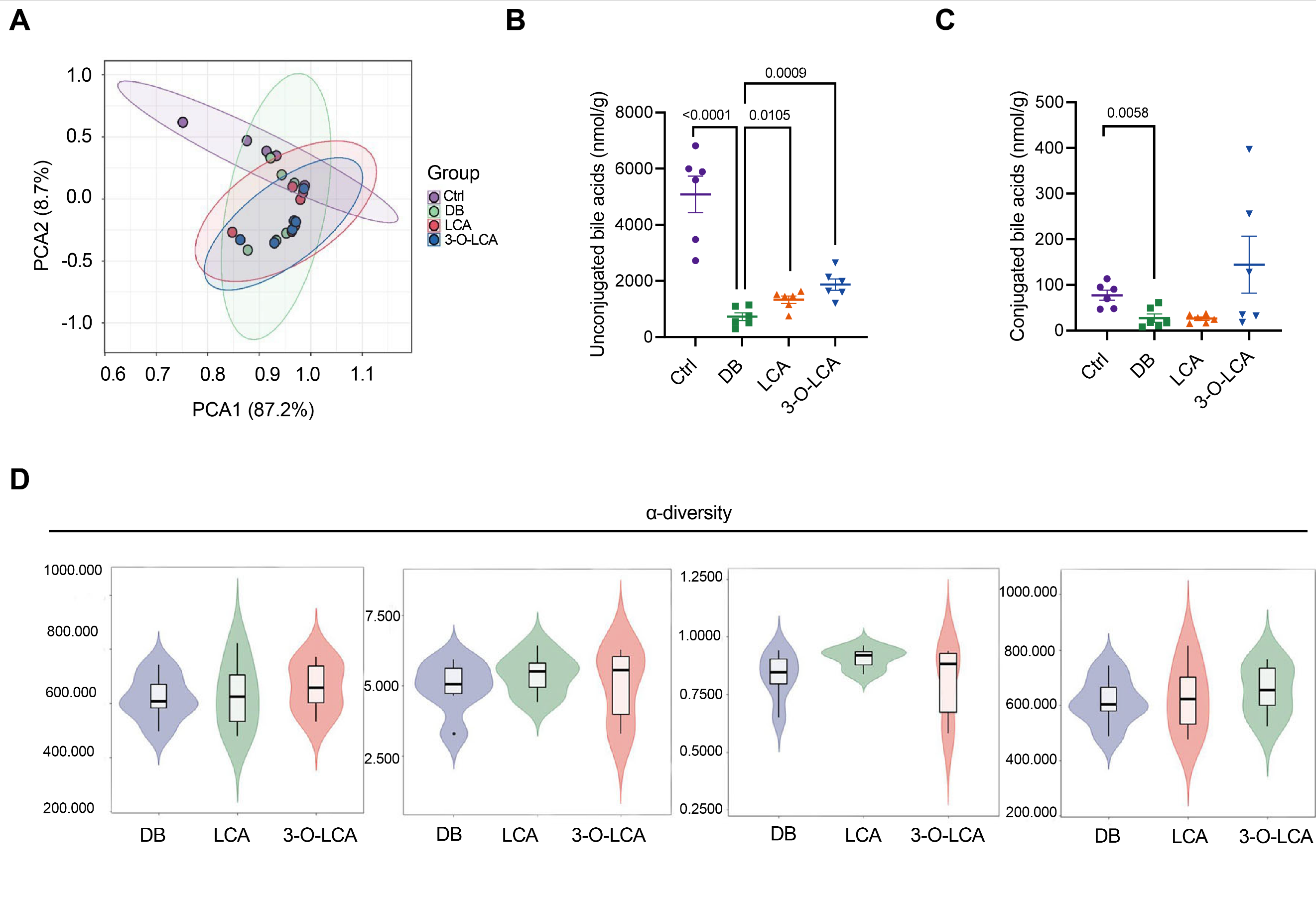


**Figure S5. The administration of LCA and 3-O-LCA ameliorated the bile acids metabolism and increased the BSH-generating microbes in eSC-IBD mice.** (A) The PCA plot revealed a distinct separation among the Ctrl group**,** DB group and the bile acid-treated groups. (B) The content of unconjugated bile acids in each group was depicted. (C) The content of conjugated bile acids in each group was depicted. (D) Alpha diversity analysis, including Chao1, Observed_otus, Shannon, and Simpson indices, was conducted on intestinal contents obtained from the three groups. All data were represented as the mean ± SEM, *p* values were calculated using one-way ANOVA.

**
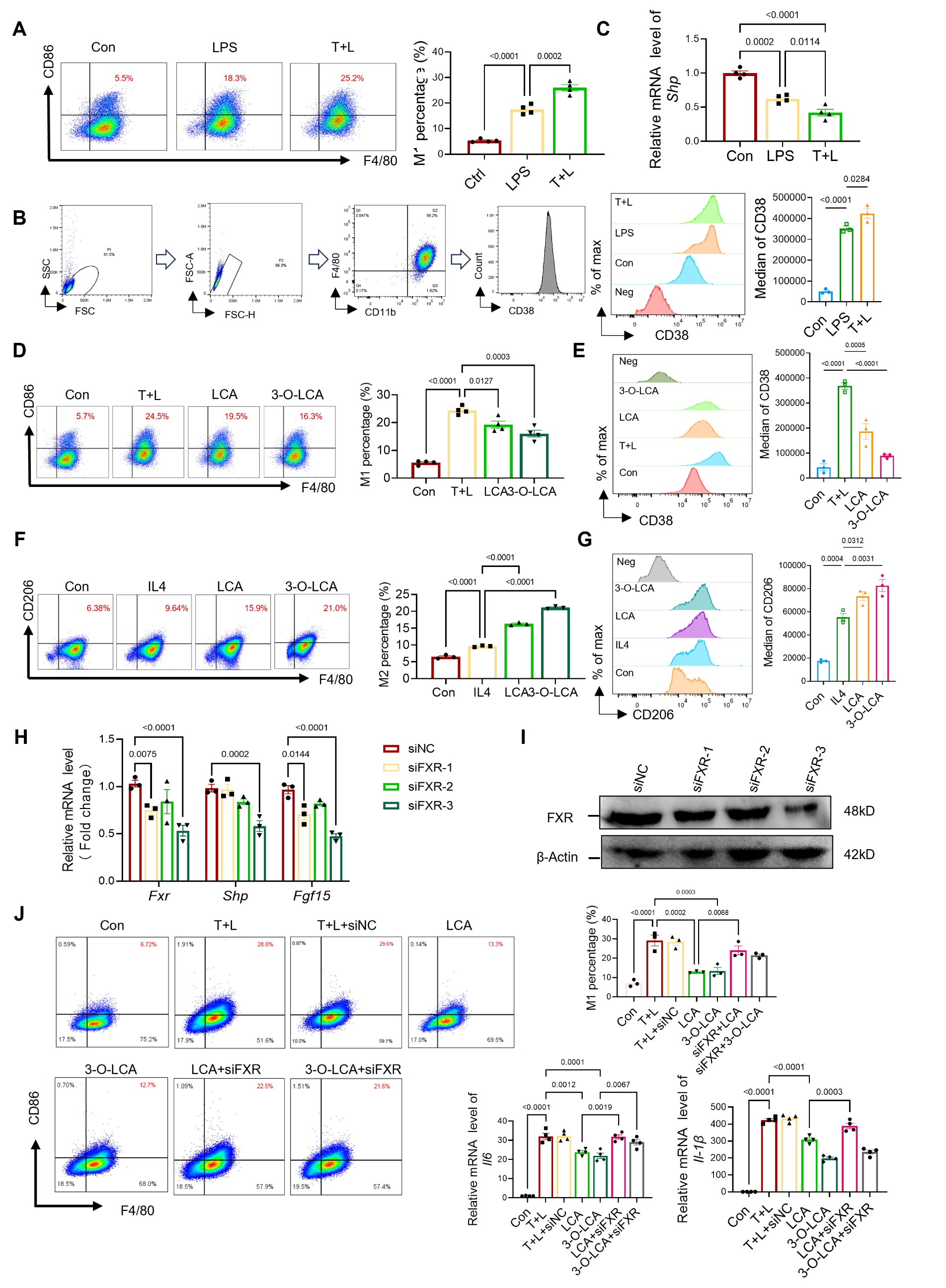
**

**Figure S6. LCA and 3-O-LCA regulated the macrophage polarization through activation of FXR.** (A) Representative flow cytometry plots showing the proportions of M1 (F4/80^+^CD86^+^) macrophages (n = 3). RAW264.7 cells were pretreated with T-β-MCA or DMSO, followed by stimulation with LPS (300 ng/mL). (B) Gating strategy for BMDM polarization analysis and flow cytometric histogram of CD38^+^ BMDMs pretreated with DMSO or T-β-MCA (25 μM for 4 h), n=3. (C) After stimulated with LPS or T-β-MCA, RAW264.7 cells were subjected to RNA isolation and the mRNA level of *shp* was presented (n=4). (D) Representative flow cytometry plots showing the effect of LCA and 3-O-LCA on M1 (F4/80^+^CD86^+^) macrophages, n=4. (E) Flow cytometric histogram of CD38^+^ BMDMs pretreated with LCA or 3-O-LCA (20 μM for 4 h), n=3. (F) The frequency of macrophage M2 polarization of RAW264.7 cells treated with LCA or 3-O-LCA were assayed via FACS analysis, n=3. (G) Flow cytometric histograms of CD206 expression in BMDMs treated with 20 μM LCA or 3-O-LCA for 24 h, n=3. (H, I) The interference efficiency of three FXR siRNAs were presented and siFXR-3 was selected for subsequently analysis, n=3. (J) Representative FACS diagram demonstrating the reduced anti-inflammatory activity of LCA and 3-O-LCA after siFXR treatment, along with the mRNA expression levels of *Il6* and *Il-1β*. All data were represented as the mean ± SEM, *p* values were calculated using one-way ANOVA. **Table S1. Primer Sequences for Real-time polymerase chain reaction.**

| **Genes** | **Forward Primer** | **Reverse Primer** |
| --- | --- | --- |
| *Il6* | ACAAAGCCAGAGTCCTTCAGA | GCCACTCCTTCTGTGACTCC |
| *Il-1β* | GAAATGCCACCTTTTGACAGTGA | TGTGCTGCTGCGAGATTTGA |
| *Il17a* | TACCTCAACCGTTCCACGTC | TTCCCTCCGCATTGACACAG |
| *Il10* | GCATGGCCCAGAAATCAAGG | TTCATGGCCTTGTAGACACC |
| *Tnfα* | AGGCACTCCCCCAAAAGATG | CCACTTGGTGGTTTGTGAGTG |
| *Mcp1* | CCTGCTGCTACTCATTCACCA | ATTCCTTCTTGGGGTCAGCA |
| *Tgf-β1* | CATCCATGACATGAACCGGC | GAAGTTGGCATGGTAGCCCT |
| *Col1a1* | GCTCCTCTTAGGGGCCACT | CCACGTCTCACCATTGGGG |
| *Col1a2* | GTAACTTCGTGCCTAGCAACA | CCTTTGTCAGAATACTGAGCAGC |
| *Cox2* | CTTCGGGAGCACAACAGAGT | AAGTGGTAACCGCTCAGGTG |
| *Cd4* | AACCAGACAGTGTTCCTGGC | ACTGGCAGGTCTTCTTCTCAC |
| *Cd8* | AGAGACCAGAAGATTGTCGGC | AACACGCTTTCGGCTCCTG |
| *Ostα* | CTGAAGGACACCCCGATGAG | TGTTGATCCAGAGAGCTGCG |
| *Ostβ* | CACCAGAAGACCTGCATCTTG | CTCTCTGTTTCCTGTGGGTCTG |
| *Mrp2* | CCTGGAAATCACGATGGACGA | AACACCTGCTTGGCAAGGTA |
| *Mrp3* | TGAGGATGCGGTCCTACTGA | GGCCGTGGGGTCAGAAATAA |
| *Mrp4* | CCTGGAATCCACAACACGGA | GTCTTCGCCAGAACAAGGGA |
| *Cyp7a1* | ATCTGGGGGATTGCTGTGGT | CCAGGTATGGAATCAACCCGT |
| *Cyp27a1* | CACAGGAGAGTACGGAGGGTC | AAGTCCCAAAGGAGGTTGTCC |
| *Ntcp* | CACAACGTATCAGCCCCCTT | GGCTTCCAGAAGTGAGCCTT |
| *Oatp1* | CAGGCACATTTACCTGGGGT | GTCTGGAGAGTGGATGTCGC |
| *Asbt* | CCATGGGGTATCTTCGTGGG | GTTCCCGAGTCAACCCACAT |
| *Bsep* | GCTGCCAAGGATGCTAATGC | TTGGGTTTCCGTATGAGGGC |
| *Acta2* | GTACCACCATGTACCCAGGC | GCTGGAAGGTAGACAGCGAA |
| *Timp1* | TGGCATCTGGCATCCTCTTG | ACCGGATATCTGCGGCATTT |
| *Ck19* | CAGTCCCAGCTCAGCATGAA | GGGGTGGGCAGATTGTTGTA |
| *Ccl3* | CCAAGTCTTCTCAGCGCCA | CCAGGTCTCTTTGGAGTCAGC |
| *Ccl7* | AGGATCTCTGCCACGCTTCT | ACACCGACTACTGGTGATCC |
| *Cxcl1* | ACTCAAGAATGGTCGCGAGG | ACTTGGGGACACCTTTTAGCA |
| *Cxcl3* | ACCCTACCAAGGGTTGATTTTG | TGGCTATGACTTCTGTCTGGGT |
| *Cxcl5* | TGCCCTACGGTGGAAGTCAT | TGCGAGTGCATTCCGCTTA |
| *Fxr* | TCGTTCGGCGGAGATTTTCA | CCGCCTCTCTGTCCTTGATG |
| *Shp* | GTACCTGAAGGGCACGATCC | CCAGGGCTCCAAGACTTCAC |
| *Il23* | TGGAGCAACTTCACACCTCC | GGCAGCTATGGCCAAAAAGG |
| *Rorc* | TGCGACTGGAGGACCTTCTA | AGACTGTGTGGTTGTTGGCA |
| *Ifng* | TGGCTGTTTCTGGCTGTTACT | CATCCTTTTTCGCCTTGCTGT |
